# Pentafluorosulfanyl-functionalised BODIPY push–pull dyes for p-type dye-sensitized solar cells†

**DOI:** 10.1039/d2se00977c

**Published:** 2023-02-21

**Authors:** Richard D. James, Linah S. Alqahtani, John Mallows, Heather V. Flint, Paul G. Waddell, Owen J. Woodford, Elizabeth A. Gibson

**Affiliations:** a Energy Materials Laboratory, Chemistry, School of Natural and Environmental Science, Newcastle University Newcastle upon Tyne NE1 7RU UK; b Department of Chemistry, College of Science, King Faisal University P.O Box 400 Al-Ahsa 31982 Saudi Arabia elizabeth.gibson@newcastle.ac.uk

## Abstract

We report a push–pull BODIPY-based dye functionalised with an electronegative SF_5_ group at the *meso* position for applications in photocathodes in tandem dye-sensitized solar cells (DSSCs). The push–pull character enhances charge-transfer from the mesoporous NiO cathode surface towards the redox mediator. A Knoevenagel condensation reaction was used to introduce the carboxylic acid to anchor the dye to the oxide surface, *via* a styryl linker which increases the conjugation in the molecule and shifts the absorption to the red. The room-temperature synthesis and high yields, make the dye promising for manufacture on a large scale. The dye was applied in p-DSSCs giving a power conversion efficiency (0.066%), a short circuit photocurrent (*J*_SC_) of 3.84 mA cm^−2^, open circuit voltage (*V*_OC_) of 58 mV and fill factor of 30%.

## Abstract 2

We report a push–pull BODIPY-based dye functionalised with an electronegative SF_5_ group at the *meso* position for applications in photocathodes in tandem dye-sensitized solar cells (DSSCs).

## Graphical abstract

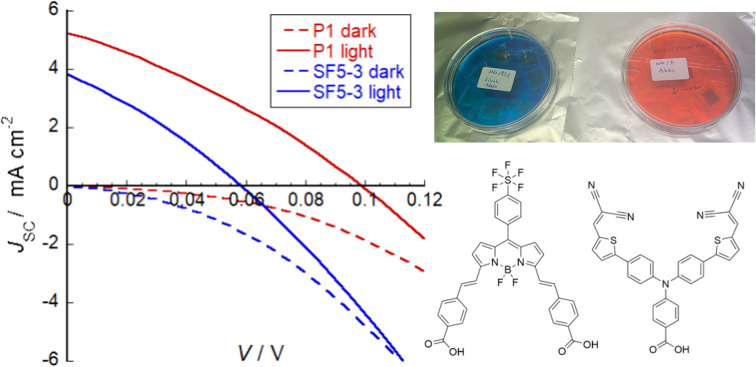

## Introduction

Since Grätzel and O'Regan's seminal paper in 1991, there has been a huge scientific effort to increase the efficiency and stability of dye-sensitized solar cells (DSSCs) to compete with established silicon photovoltaics.^1^ DSSCs perform very well in diffuse light conditions, they have variable colours, shapes and transparency and they can be printed on lightweight substrates like foils or plastic as well as glass, providing extensive opportunities for integration into building materials or electronic devices and new markets where silicon technology is unsuitable.^2,3^ The principal components of a DSSC include the dye, electrolyte, semiconductor, and counter electrode. The dyes absorb photons of specific wavelength and inject the resulting excited electrons into the conduction band of the semiconductor to cause charge to flow to the substrate and generate a photocurrent. A redox electrolyte regenerates the ground state of the dye by shuttling electrons from the counter electrode to reduce the oxidised dye molecules. The voltage is provided by the difference in the electrochemical potential of the redox electrolyte and the quasi-Fermi level of electrons in the conduction band of TiO_2_. Efforts to improve efficiency by extending the spectral range of the dye to increase the photocurrent typically led to a lower voltage. Efforts to increase the conduction band edge of the semiconductor and increase the voltage, often lead to a loss in photocurrent. For these reasons, the overall efficiency has only risen incrementally in the last 10 years.

**Fig. 1:**
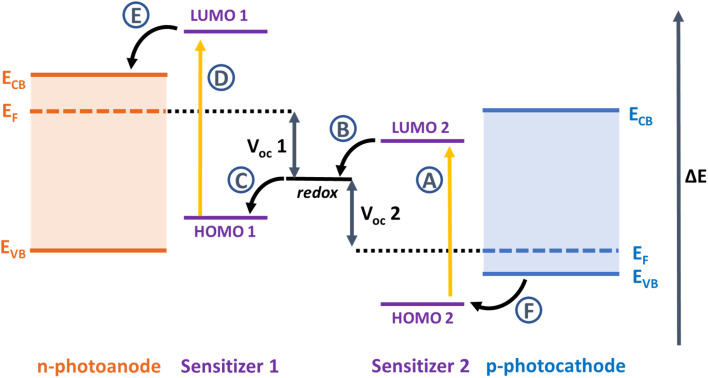
Relative energy level diagram and the components in a tandem dye-sensitized solar cell. The maximum *V*_OC_ of the tandem device should be the sum of the *V*_OC_ of the individual single junction cells (*V*_OC_ 1 and *V*_OC_ 2). A–F are electron transfer processes following absorption by light: A and D are photoinduced excitation of sensitizers 2 and 1, B and D are dye-regeneration processes at the dye-electrolyte interfaces, and E and F are charge-injection processes at the dye-semiconductor interfaces.

An alternative approach is to develop a tandem dye-sensitized solar cells, where the cathode is replaced by a photoactive electrode (Fig. 1).^4–6^ If the dyes used in the photocathode harvest the longer wavelengths (>700 nm) that are transmitted by the TiO_2_-based photoanode, then more light can be converted, more efficiently.^7^ Alternatively, the photocathode could be sensitized with a dye absorbing shorter wavelengths as used as the top electrode and an NIR dye could be used to sensitize the photoanode at the bottom.^8^ As with conventional DSSCs, the criteria for the dye are a high molar absorption coefficient and suitable redox potentials which straddle the valence band of the p-type material in the photocathode and the redox potential of the electrolyte to drive charge separation. Much work has been devoted to developing dyes for TiO_2_. Relatively little by comparison has been invested in dyes for photocathodes, where the charge moves in the opposite direction to photoanodes.^9^

The dyes that have been applied in p-DSSCs include perylene diimides, squaraines, diketopyrrolopyrrole, quinones, pyran-based systems, porphyrins, triphenylamine donor-π linker – acceptor structures, and combinations of these.^2,3,8^ The push–pull dye P1 is generally considered to be a good benchmark, due to optimised short circuit current density (*J*_SC_) = 5.47 mA cm^−2^, incident photon to conversion efficiency (IPCE) = *ca.* 63%, and power conversion efficiency (PCE) of 0.16% with I_3_^−^/I^−^ electrolyte. A higher *J*_SC_ was attained by switching the nitrile acceptor for indoliums and blue-shifting the absorption in the CAD3 dye (*J*_SC_ = 8.21 mA cm^−2^, IPCE = 50%, PCE = 0.25). Bach *et al.* achieved some of the highest performances for p-DSSCs using the dye PMI-6T-TPA (*J*_SC_ = 6.26 mA cm^−2^, IPCE = 52%, PCE = 0.60%).^10^ The device efficiency was increased to 2.51%, the current highest PCE reported for p-DSSCs, by substituting the I_3_^−^/I^−^ redox shuttle for [Fe(acac)_3_]^0/1−^ (*J*_SC_ = 7.65 mA cm^−2^, IPCE = 57%). The tandem solar cell efficiency reached for this dye and I_3_^−^/I^−^ electrolyte was 2.4%.^11^ This was surpassed by Odobel *et al.* with a diketopyrrolopyrrole dye, Th-DPP-NDI (*J*_SC_ = 6.73 mA cm^−2^, *V*_OC_ = 910 mV, PCE = 4.1%).^7^ The champion photocathode produced a *J*_SC_ = 8.2 mA cm^−2^, IPCE = 57% and PCE = 0.44%.

In our research group, we have been investigating the properties of 4,4-difluoro-4-bora-3*a*,4*a*-diaza-*s*-indacene (BODIPY) photosensitizers for NiO based photocathodes, because they resist photobleaching, have high molar absorption coefficients, and their optical and electronic properties can be easily tuned through simple structural modifications.^12–17^ DFT modelling of the dyes reported previously, with benzoic acid anchoring groups at the *meso* position, showed that in the ground state the BODIPY chromophore is electronically decoupled from the NiO surface and in the excited state, electron density is pushed towards the NiO rather than towards the electrolyte.^18^ We have also learned that it is extremely important to incorporate a “push–pull” structure to drive charge transfer away from the photocathode surface towards the electrolyte.^17^ This was achieved by linking a triphenyl amine-based electron donating group, with carboxylic acid groups to bind to the metal oxide surface, to the BODIPY *via* thiophene π-linker to the BODIPY chromophore. The champion dye GS1 sensitized p-DSSCs had a *J*_SC_ = 5.87 mA cm^−2^, IPCE = 53% and PCE = 0.20%.^12^ In 2019, Kubo *et al.* produced a red-shifted BODIPY, with anchoring groups at the 3,5 positions.^19^ Extended pi-systems were added to the β-pyrrole positions to push the absorption into the NIR (<830 nm). However, overall device efficiencies were very low (open circuit voltage *V*_OC_ = 79 mV, *J*_SC_ = 0.61 mA cm^−2^, fill factor (FF) = 0.25, and PCE = 0.012%), due to rapid recombination between the reduced dye and the NiO surface. In order to overcome this issue, new electron acceptor groups could be used upon the BODIPY core moiety to improve charge separation within the dye. One group that could fulfil this role is the pentafluorosulfanyl acceptor.

Sheppard *et al.* first created the pentafluorosulfanyl moiety in the 1960s,^20^ yet the hazardous synthetic pathways and poor availability of the group commercially meant that its applications were not thoroughly explored. Recently, due to an increase in material availability, there has been an increase in research into the group for applications in such areas as medicinal chemistry, agrochemicals, liquid crystals, and energy research.^21–27^ The SF_5_ group boasts good chemical and thermal stability, large steric bulk, and electron withdrawing character. These properties are useful for applications in dye-sensitized solar cells, however the SF_5_ group has not been reported for this application so far. Based on our previous work, we have re-designed the BODIPY dye system to incorporate SF_5_ at the *meso*-aryl position and appended from the pyrrole styryl groups with carboxylic acid groups to bind the dye to the NiO surface.

## Results and discussion

### Dye synthesis and properties

**Fig. 2:**
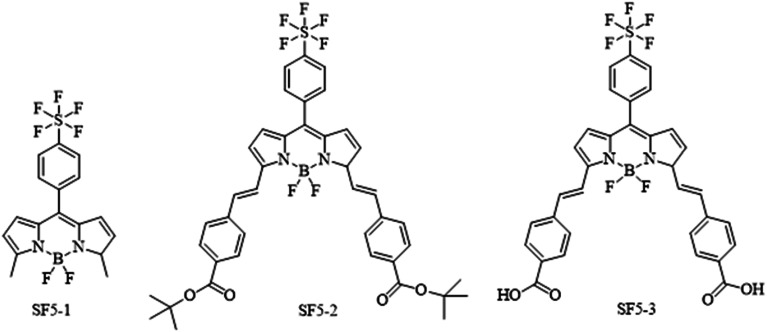
Chemical structures of the dyes SF5-1–3 synthesised and tested in this work.

Fig. 2 shows the structures of the three BODIPY compounds produced in this work. The experimental details, synthetic routes and characterisation data can be found in the ESI.† Briefly, for SF5-1, the precursor 2-methyl pyrrole was obtained *via* previously reported procedure.^28^ This was reacted with 4-(pentafluorosulfanyl)benzaldehyde in an acid catalysed condensation reaction, followed by oxidation with *p*-chloranil. The subsequent deprotonation and chelation steps were performed using diisopropylethylamine and borotrifluoroetherate respectively. The product SF5-1 was purified *via* column chromatography, made straightforward by the high steric bulk of the SF_5_ group that reduced π–π stacking, leading to good solubility of the molecule.

To prepare SF5-2, piperidine and acetic acid were used to catalyse a room-temperature Knoevenagel condensation with *tert*-butyl 4-formylbenzoate over 4 hours. This unconventional room temperature synthesis can be achieved because of the reduced steric hindrance at the BODIPY *meso* position, and the beneficial electronegativity of the SF_5_ moiety. An analogue with only one styryl arm on the α position was observed as an intermediate which was separated from the primary product by thin layer chromatography. This was not isolated as the intermediate outcompetes the starting material in terms of reactivity and the condensation occurs preferentially on the intermediate. This resulted in a high-yielding reaction with only trace quantities of unreacted intermediate. Purification using column chromatography led to hydrolysis of SF5-2 by the acidic silica, unless it was completed within two hours. Basifying the column with triphenylamine improved mobility and eliminated this problem. With the general molecular structure established, the addition of carboxylic acid anchoring groups was necessary to bind the molecule to the NiO surface.

Acid hydrolysis of SF5-2 was performed by stirring overnight with excess tetrafluoroacetic acid to give quantitative yields of SF5-3. The poor solubility of SF5-3 meant that purification by column was unsuitable, but unnecessary as the product spontaneously precipitated. All three dyes were characterised by NMR and mass spectrometry to confirm their structure.

### Crystal structure

**Fig. 3:**
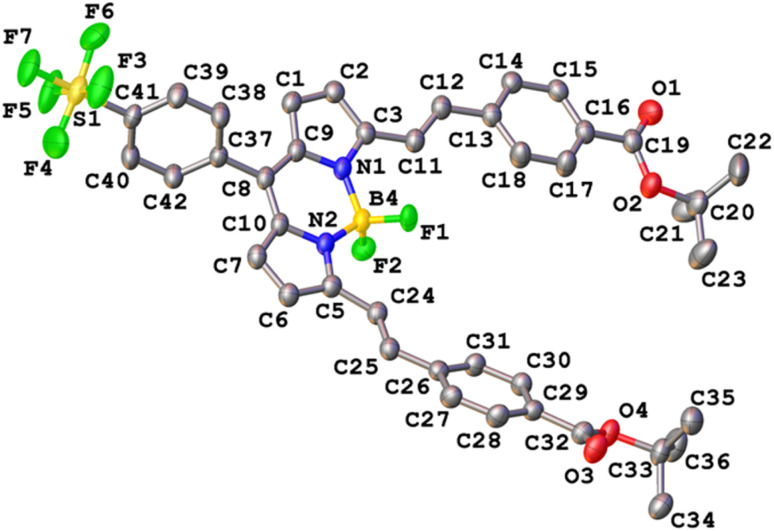
Crystal structure of SF5-2. Thermal ellipsoids drawn at 50% probability.

Crystals of SF5-1 and SF5-2 suitable for single-crystal X-ray diffraction analysis were grown from a mixture of dichloromethane and hexane. The structure of SF5-1 is shown in Fig. S1 (ESI†) and the structure of SF5-2 is shown in Fig. 3. Both molecules are almost planar, demonstrating the conjugated electronic structure that is consistent with the DFT calculations (below). The torsion angles are less than 16 Å at either side of the alkene, giving a small twist (10 and 20 Å for styryl at the 3 and 5 positions on the BODIPY, respectively). The torsion angle between the styryl bridge and the carbonyl of the anchoring group is only 7 (C11–C23) and 13 (C24–C36) Å. This structure should facilitate electron transfer from the NiO, through the anchoring group and styryl linker to the BODIPY core.

### Computational analysis

Previously we found that placing a benzoic acid anchoring group at the *meso* position was unfavourable for charge-separation in p-DSSCs because in the ground state there is a node on C-8.^14,15^ The electron density of the LUMO, however, spreads onto C-8 and aromatic substituents at the *meso* position. Kubo positioned a triarylamine carboxylic acid anchoring group at the C-3 and C-5 positions, expanding the electron density of the HOMO over the anchoring group and BODIPY chromophore. Electron withdrawing substituents were positioned at C-1 and C-7 so that the LUMO was directed towards the opposite side of the molecule, away from the anchoring groups. Adding styryl groups at C-3 and C-5 is known to shift the absorption spectrum of BODIPY dyes to longer wavelength through extension of the conjugation, and this was observed experimentally for SF5-2. This structure should enable conjugation of the BODIPY core through to the carboxylic acid anchoring groups in SF5-3 and promote charge transfer from NiO towards the electron withdrawing substituent at C-8.

**Fig. 4:**
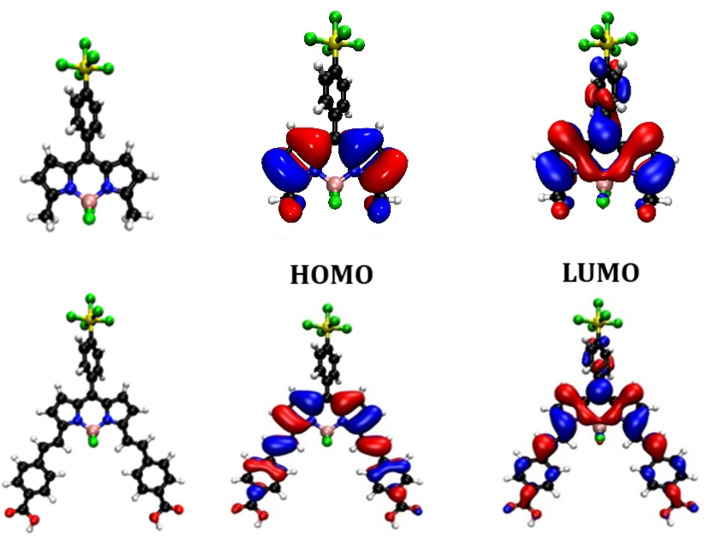
Optimised geometry and orbital distribution (left = HOMO, right = LUMO) for SF5-1 (top) and SF5-3 (bottom) in MeCN using cam-B3LYP 6-311G++ (d,p).

To visualise the origin of the optical and electrochemical properties of these new dyes, density functional theory calculations were employed. The theoretical energy levels and corresponding molecular orbital distributions were calculated in vacuum using cam-B3LYP 6-311G ++ (d,p), and in MeCN using a polarisable continuum solvent model (IEFPCM). TDDFT calculations were then used to determine the vertical excitation energies. Only the singlet excited states were considered. The HOMO-LUMO orbital distributions are shown in Fig. 4 and the calculated energy transitions are tabulated in the ESI.† The predicted energy transitions and oscillator strengths correspond well with the experimentally observed wavelengths and relative intensities of the peaks, albeit hypsochromically shifted.

The calculated lowest energy excitation for both SF5-1 and SF5-3 corresponded with a HOMO to LUMO π–π* transition. For SF5-1, the electron density of the HOMO was found to be distributed over the BODIPY core and aryl ring, whereas the electron density of the LUMO was localised on the aryl-SF_5_ group. For SF5-3, the planar extended structure, with the aryl rings of the styryl groups located in line with the BODIPY core, led to a broader distribution of electron density in both the HOMO and LUMO, which included the carboxylic acid anchoring groups. This electronic distribution is consistent with the bathochromic shift of the absorption spectrum of SF5-2 and SF5-3 compared to SF5-1. The first excited state is therefore associated with the π–π* transition from the HOMO to LUMO and has some charge transfer character towards the aryl group in the *meso* position of the BODIPY.

### Optical properties

**Fig. 5:**
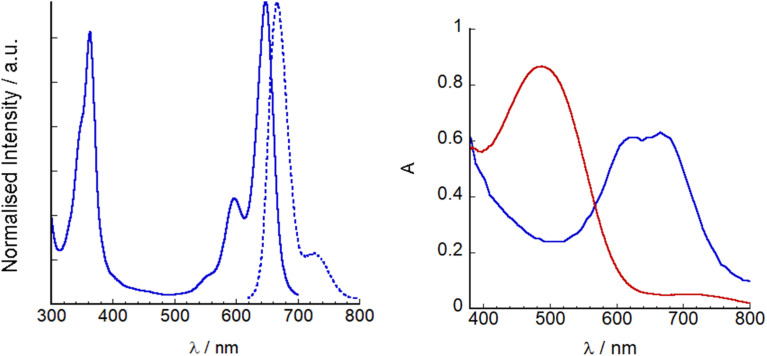
Left: UV-visible absorption (line) and emission spectra (*λ*_ex_ = 620 nm, dashed line) of SF5-3 in THF. Right: UV-visible absorption spectra of P1|NiO (red) and SF5-3|NiO (blue).

The UV-visible absorption and fluorescence spectra of SF5-1 and SF5-2 are shown in Fig. S4† and a summary is provided in Table 1. The absorption maxima at 511 nm and 642 nm for SF5-1 and SF5-2 respectively were assigned to the S^0^ → S^1^ (π → π*) transition, with a red shift of 131 nm upon addition of the extended styryl moieties. Both also display a vibrational shoulder, with the SF5-1 peak diminished in comparison to SF5-2. The molar absorption coefficient SF5-2 (79 400 M^−1^ cm^−1^) was much higher than that of the benchmark dye P1 (66 000 M^−1^ cm^−1^) and our previous champion BODIPY dye, GS1, (65 700 M^−1^ cm^−1^)^17^ making it a promising photosensitizer. The absorption maxima of SF5-2 and SF5-3 were also significantly red shifted compared to P1 and GS1 (565 nm) but not as much as the dye reported by Kubo *et al.* (*λ*_max_ = 730 nm).^12^ The higher energy band observed at around 350 nm for both SF5-1 and SF5-2 is characteristic for BODIPY molecules and has been assigned as an S^0^ to S^2^ peak. The emission spectra of both dyes have a small Stokes shift, characteristic of BODIPY compounds. SF5-2 had a higher fluorescence quantum yield and lifetime compared to SF5-1 which we attribute to the more rigid structure of SF5-2. Fig. 5 shows the absorption and emission spectra of SF5-3 in THF as the dye was only sparingly soluble in MeCN. There is little change between the spectra for SF5-3 and SF5-2 except for a small red-shift, showing that the electronic properties are largely unchanged on hydrolysis. The quantum yield for fluorescence was slightly lower for the acid SF5-3 compared to the ester SF5-2, however the fluorescence emission lifetime we recorded was almost the same (Table 1).

**Table 1:** Summary of optical and electrochemical properties of SF5-1 and SF5-2 MeCN, and SF5-3 in THF^a^

Dye	*λ*_max_ (nm)	*ε* (M^−1^ cm^−1^)	*E*_00_ (eV)	*Φ*	*τ* (ns)	*E*_1/2_ [D^+^|D] (V *vs.* Fc)	*E*_1/2_ [D|D^−^] (V *vs.* Fc)
P1 (ref. 29)	481	57 000	2.25		0.03	0.69	−1.46
SF5-1	511	69 500	2.37	0.11	0.54	0.64	−1.24
SF5-2	642	79 400	1.89	0.57	4.4	0.68	−0.97
SF5-3	648	71 200	1.89	0.37	4.5	0.90	−1.17

a *λ*_max_ is the wavelength of maximum absorption; *E*_00_ is the zero–zero energy determined from the intersection of the normalised absorption and emission spectra; *Φ* and *τ* are the fluorescence quantum yield and lifetime, respectively, which were recorded with an excitation wavelength of 505 nm (SF5-1) and 635 nm (SF5-2 and SF5-3); *E*_1/2_ [D^+^|D] and *E*_1/2_ [D|D^−^] are the ground state half potentials for oxidation and reduction of the dye measured in solution with 0.2 M TBAPF_6_, glassy carbon working electrode and calomel reference electrode and calibrated against Fc.

SF5-3 and P1 were adsorbed on NiO from MeCN solution (*ca.* 0.3 mM). Dye loading studies (see Experimental section for details) gave 14 nmol cm^−2^ for P1 and 11 nmol cm^−2^ for SF5-3, consistent with the larger footprint of SF5-3 compared to P1 with the single-anchoring group. Therefore, there appears to be a trade-off between absorption coefficient and dye-loading.

### Photophysics

**Fig. 6:**
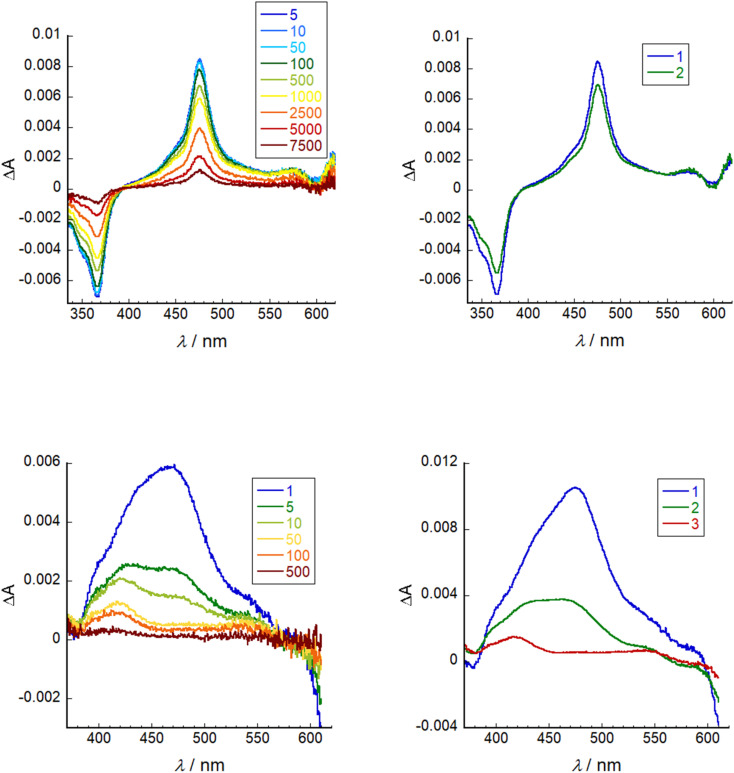
Left: transient absorption spectra (*λ*_ex_ = 640 nm) of SF5-3 in THF (top) and SF5-3|NiO (bottom). Right: evolution associated spectra of SF5-3 in THF where 1 = 260 ps and 2 = 4.0 ns (top) and SF5-3|NiO where 1 = 0.87 ps, 2 = 8.3 ps, 3 = 230 ns (bottom).

Transient absorption spectroscopy was conducted on a solution of SF5-3 in THF and SF5-3 adsorbed on the surface of a mesoporous NiO electrode (SF5-3|NiO), excited close to the maximum absorbance at 640 nm. Global analysis was performed using Optimus. The spectra and fitting is shown in Fig. 6. In solution, the excited state decay required two lifetimes, *τ*_1_ = 260 ps and *τ*_2_ = 4.0 ns, which is in agreement with the fluorescence lifetime and quantum yield. When adsorbed on NiO, the excited state absorption was broader than in solution, consistent with the broader ground state absorption. The first component had an absorption maximum close to that of the excited state in solution and a lifetime of *τ*_1_ = 0.87 ps. This suggests that the excited state decays rapidly (<1 ps) when the dye is adsorbed on NiO. The third component was relatively long lived (*τ*_3_ = 230 ns) and contains a peak that was blue shifted relative to the excited state transient (*λ*_max_ = *ca.* 420 nm) and a broad, featureless absorption that is consistent with oxidised NiO.^30^ The second component (*τ*_2_ = 8.3) has features that are a combination of the first and third species and is consistent with the characteristically heterogeneous behaviour of dyes on metal oxide surfaces, representing a slightly slower injection process and a rapid charge-recombination process.^31^ In our previous work, we found that when the BODIPY was anchored through the *meso* position, charge-recombination was complete within 1 ns.^15,32^ By anchoring through the pyrrole, the charge-separated state lifetime has been increased by *ca.* 3 orders of magnitude. This lifetime is longer than the previous longest-lived BODIPY radical anion on NiO observed by our group (*τ* = 180 ns).^13^

### Electrochemistry

The redox potentials of the dyes are summarised in Table 1 and the data is shown in the ESI.†SF5-1 undergoes three processes, one pseudo-reversible reduction (*E*_1/2_ [D|D^−^]) another non-reversible reduction, and an oxidation (*E*_1/2_ [D^+^|D]). Based on the DFT calculations for the electronic distribution in the LUMO, the first process is expected to be a BODIPY-centred reduction and *E*_1/2_ [D|D^−^] was similar to GS1.^12^ The second irreversible reduction is attributed to reduction of the aryl-pentafluorosulfanyl group, based on the electronic distribution in LUMO+1 calculated using DFT, and irreversible reductions of –SF5 substituents been reported previously.^33^ Upon addition of the styryl groups, *E*_1/2_ [D|D^−^] of SF5-2 was shifted to positive potential compared to SF5-1 and previously reported BODIPY dyes for p-DSSCs such as GS1. The unsubstituted 1,7 and 2,6 positions make the BODIPY core susceptible to oxidative reaction in both molecules. There was little difference in *E*_1/2_ [D^+^|D] for SF5-1 and SF5-2 and the values were similar to our previously reported BODIPY dyes GS1 and BOD1-3 (0.62–0.69 V *vs.* Fc) and P1 but more positive than the expanded BODIPY dye by Kubo *et al.*^12,15,16^ This suggests that the addition of the styryl group to the pyrrole groups has more of an electronic effect on the potential energy of the LUMO than the HOMO, which we found surprising.

The cyclic voltammetry of SF5-3 was conducted using THF as the solvent, because of the limited solubility of the acid in MeCN. For this dye, the oxidation was more positive than the ester (0.90 V *vs.* Fc) and the first and second reduction processes were more negative (−1.17 V and −1.66 V *vs.* Fc). The driving force for charge-separation should be similar for the P1 and SF5-3 dyes (*ca.* 65 meV greater for P1 than SF5-3) and compared to those previously reported. However, the driving force for dye-regeneration should be substantially smaller (*ca.* 290 meV). An advantage could be that less energy is consumed in the dye-regeneration step, allowing more lower energy photons to be harvested.

### p-Type photocathodes and solar cells

**Fig. 7:**
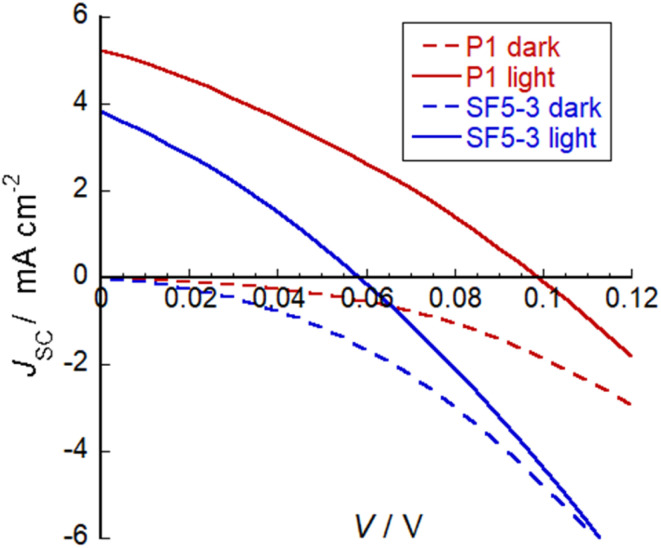
Plot of current density against voltage for illuminated devices containing dye SF5-3 and the benchmark dye P1. Electrolyte composition: 0.1 M LiI and 1 M I_2_ in MeCN.

SF5-3 was applied NiO-based p-type DSSCs and current–voltage curves were obtained in both the dark and under AM1.5 G, 100 mW cm^−2^ illumination. Fig. 7 shows the current–voltage plots and the promising performance of SF5-3 as a blue-coloured dye for photocathodes. A summary of the device characteristics is provided in Table 2. For the optimised devices, the current density (*J*_SC_) and open circuit voltage (*V*_OC_) for SF5-3 was *ca.* 27% lower than the device containing the benchmark dye P1. This is consistent with the *ca.* 25% lower dye-loading of SF5-3 compared to P1. The dark current measurement indicated that there recombination at the electrode/electrolyte interface greater for the SF5-3 dye, possibly due to interactions between the dye and the iodine-based electrolyte. The lower photocurrent density observed for SF5-3 compared to P1 could also be partly attributed to the smaller driving force for regeneration (Δ*G* = −350 meV for SF5-3 compared to −640 meV for P1).

**Table 2:** Characteristics of champion p-type dye-sensitized solar cells. Open circuit voltage (*V*_OC_), short circuit current (*J*_SC_), fill factor (FF) and efficiency (*η*) of NiO p-DSSCs based on dyes SF5-3 and P1

Dye	*V*_OC_ (mV)	*J*_SC_ (mA cm^−2^)	FF (%)	*η* (%)	Dye loading nmol cm^−2^
SF5-3	58	3.84	30	0.066	14
P1	98	5.26	31	0.16	11

The performance of SF5-3 as a photosensitizer for p-type DSSCs was promising compared to previously reported BODIPY dyes. The change in structure led to an enhancement in the photocurrent compared to BOD1-3 (*J*_SC_ < −0.6 mA cm^−2^).^15^ This demonstrates the advantages of the increased conjugation between the core and the anchoring group, the electron accepting group at the *meso* position and the red-shifted absorption in enhancing the solar cell performance. The photocurrent density with SF5-3 was not as high as that attained with our champion push–pull BODIPY dye GS1 (*J*_SC_ = 5.87 mA cm^−2^, IPCE = 57%), despite the red-shifted absorption spectrum.^12,14^ It was, however, substantially higher than the photocurrent density reported by Kubo *et al.* for the expanded, NIR-absorbing BODIPY (*J*_SC_ = 0.61 mA cm^−2^, IPCE = 3%).^16^ Kubo *et al.* suggested that their system may be limited by rapid recombination, but it may also be due to a sub-optimal offset between the electronic states in the dye and the NiO valence band leading to inefficient charge-separation.^34^

## Conclusions

The application of NiO electrodes into tandem devices calls for the creation of red shifted absorbers to harness the breadth of the solar spectrum. These absorbers require strong binding groups and extended charge separated lifetimes to reduce recombination and increase current flow within the device. Three BODIPY dyes were developed which contained a strongly electronegative SF_5_ group in the *para* position of the *meso*-aryl group. The molecules were synthesised using room-temperature procedures in high yields, making them excellent candidates for scaling up. The chromophore SF5-3 contained a carboxylic acid anchoring group to bind to NiO photocathodes. The SF5-3-sensitized photocathode was applied in p-DSSC's which gave a power conversion efficiency of 0.066%, a promising short circuit photocurrent density (*J*_SC_) of 3.84 mA cm^−2^, open circuit voltage (*V*_OC_) of 58 mV and fill factor of 30%. The performance seems to be limited by the light-harvesting efficiency, which was lower than the benchmark P1 dye, and charge-recombination at the electrode–electrolyte interface, which was higher for SF5-3 compared to P1. The structure of the dye should be, in future, modified to raise the energy of the frontier electronic orbitals to drive charge-separation and reduce the HOMO-LUMO energy gap to shift the absorption further towards the NIR.^8^ Expansion of the BODIPY core to increase conjugation may enhance the absorption coefficient and light harvesting efficiency, enabling more photocurrent to be generated by the photocathode.^19^ Adding bulky substituents around the core may help to protect the NiO from the electrolyte and increase the voltage.^34^

## Conflicts of interest

There are no conflicts to declare.

## Supplementary Material

SE-007-D2SE00977C-s001
