# Supplementary material for: Pentafluorosulfanyl-functionalised BODIPY push–pull dyes for p-type dye-sensitized solar cells
Source: Sustain Energy Fuels. 2023 Feb 21;7(6):1494–501. doi: 10.1039/d2se00977c (PMC10013097; doi:10.1039/d2se00977c)
Supplement: SE-007-D2SE00977C-s001 [file SE-007-D2SE00977C-s001.pdf]

Electronic Supplementary Material (ESI) for Sustainable Energy & Fuels.
This journal is © The Royal Society of Chemistry 2023

SUPPLEMENTARY INFORMATION

Pentafluorosulfanyl-functionalised BODIPY push-pull dyes for p-type dye-sensitized solar cells

Richard D. James[1†], Linah S. Alqahtani[1,2†], John Mallows[1], Heather V. Flint[1], Paul Waddell[1], Owen Woodford[1], Elizabeth A. Gibson[1]*

[1]Energy Materials Laboratory, Chemistry, School of Natural and Environmental Science, Newcastle University, Newcastle upon Tyne, NE1 7RU, United Kingdom.

[2]Department of Chemistry, College of Science, King Faisal University, P.O Box 400, Al-Ahsa 31982, Saudi Arabia.

E-mail: elizabeth.gibson@newcastle.ac.uk

[†]These authors contributed equally

Experimental

Materials and Procedures

Materials were purchased from Fluorochem and Sigma unless otherwise stated

P1 dye (below) was synthesised via previous literature procedure.[1]

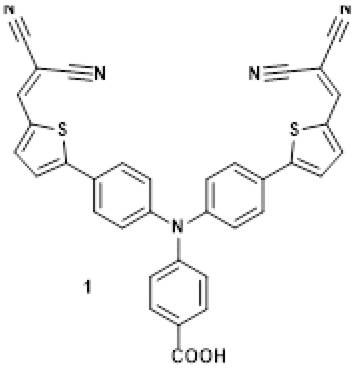

General Physical Measurements

UV-Visible absorption spectroscopy measurements were conducted on Shimadzu (UV-1800) UV-Visible spectrometer using 1 cm quartz cuvettes, 1 nm sample interval and ran in air unless otherwise stated. All Beer-Lambert measurements were kept under 1.5 absorbance units and

measured over at least five concentrations, all analytical glassware used was grade A and solvent was of HPLC grade. Absorption spectra of films was carried out using an ocean optics fibre optic setup connected to LS-1 light source and USB2000 detector.

Emission and excitation measurements were run on a Shimadzu (RF-6000) fluorescence spectrometer using 1 cm quartz cuvettes and HPLC grade solvents where appropriate. Scan speed was 600 nm/min with slits at 3.0 nm for both source and detector unless otherwise stated. Quantum yield measurements were kept under 0.1 absorbance to eliminate self-quenching and referenced vs either; **SF5-1** in MeCN (excited at 505 nm) were compared with Rhodamine 6G, excited at 490 nm (fluorescence quantum yield of 0.95 in methanol) or **SF5-2** and **SF5-2** in THF (excited at 635 nm) were compared with meso tetraphenyl porphyrin in toluene excited at 600 nm (quantum yield of 0.07, air saturated).

Photoluminescence lifetimes were determined by time-correlated, single photon counting methods (TCSPCS) using an EasyLife TCSPC unit (PTI) following excitation at either 505 nm or 635 nm with a pulsed LED (FWHM=0.35 ns). Decays were recorded using in optically thin solutions using spectroscopy grade THF as the solvent. Glass cut-off filters were used to exclude stray excitation light. Data analysis was made by standard protocols based on recursive Levenberg–Marquardt statistics.

Transient absorption spectroscopy was performed using a Helios spectrometer from Ultrafast Systems. The system utilises a Solstice Ace (Spectra physics) as the laser to generate pump and probe beams. Laser pulses are of 100 fs of 800 nm light and a repetition rate of 1 kHz. The white light continuum is generated by passing 800 nm light through a sapphire crystal. The pump light is modified to the required wavelength by an optical parametric amplifier, OPA (Apollo-T, Ultrafast Systems). The spectra are recorded by means of a fibre couple CCD array. Excitation of the samples was with depolarised light at 640 nm with 50 nJ

pump energy and a spot size of 50 μm. 3 scans were collected per sample with an integration time of 1 s at each delay time. Solution samples were stirred and delay times were randomly varied to minimise bleaching effects. Solution samples were dissolved in THF (spectroscopy grade) to an optical density of 0.4 and performed in a 2 mm path length cuvette. No visible bleaching of the films or solution samples was observed.

Global analysis was performed using the program OPTIMUS [2]. The chirp correction was applied prior to global analysis. In global lifetime analysis, a model function for each detection wavelength is constructed. The optimisation is initiated with some starting values used to calculate each of the basic functions. The amplitudes of each function are found by linear fitting using the Moore-Penrose pseudoinverse (MATLAB pinv) function. The fit parameters are optimised iteratively to minimise the square of the residual norm. The amplitudes are plotted against the detection wavelength to yield the decay associated spectra. The evolution associated spectra were determined by solving a system of differential equations using a sequential relaxation scheme. The fit parameters are optimised iteratively to minimise the square of the residual norm. The amplitudes are plotted against the detection wavelength to yield the evolution associated spectra (EAS), which represent the stationary spectra of the model compartments. For the sequential relaxation scheme, the decay associated spectra (DAS) of the lifetime components was calculated from a linear combination of the EAS of the compartments.

The electrochemical characterisation was a three-electrode set up, with a platinum counter electrode, a calomel reference, and a glassy carbon working electrode, using a tetrabutylammonium hexafluorophosphate electrolyte. The solvent was acetonitrile for **SF5-1** and **SF5-2** and tetrahydrofuran for **SF5-3**, which were degassed before each run.

Single crystal X-ray diffraction data were collected at 150 K on a XtaLAB Synergy HyPix-Arc 100 diffractometer equipped with an Oxford Cryosystems CryostreamPlus open-flow N_2

cooling device using copper X-radiation (λCuKα = 1.54184 Å). Intensities were corrected for absorption using a multifaceted crystal model created by indexing the faces of the crystal for which data were collected [3]. Cell refinement, data collection and data reduction were undertaken via the software CrysAlisPro [4]. All structures were solved using XT [5] and refined by XL [6] using the Olex2 interface [7]. All non-hydrogen atoms were refined anisotropically and hydrogen atoms were positioned with idealised geometry. The displacement parameters of the hydrogen atoms were constrained using a riding model with U(H) set to be an appropriate multiple of the Ueq value of the parent atom.

Solar Cell Assembly and Measurements

Blank fluorine doped tin oxide (FTO) glass substrates (Pilkington TEC 15 for working, TEC 8 for counter) were prepared by first cutting to size (18 x 15 mm) followed by cleaning with soapy water, 0.1 M HCl in ethanol, and lastly ethanol only. After cleaning, the glass surface was treated with ozone (using a Novascan PSD Series Digital UV Ozone System) for 15 mins before application of the electrode material. Counter electrodes were prepared by drilling a small hole (approx. 1 mm Ø) into the substrate to allow for electrolyte to be added post-assembly. Platisol-T solution (0.2 mL cm^{-2}, Solaronix) was added dropwise to the conductive surface of the glass and annealed at 450 ^{o}C for 15 mins in a Nabertherm B150 Chemical Oven to give a platinised counter electrode. Sol gel NiO precursor was prepared by dissolving anhydrous $NiCl_2$ (1 g) and a tri-block co-polymer, F108 (poly(ethylene glycol)-block-poly(propylene glycol)-block-poly(ethylene glycol) (1 g) in a mix of EtOH (6 ml) and deionised water (5 ml). The solution was left to age for 14 days and centrifuged to remove remaining solids before use. To prepare the electrodes, the doctor blade method was used to apply the precursor solution over a Scotch Magic tape spacer to form films with an active area of 0.2 cm^2. The films were sintered in a Nabertherm B150 Chemical Oven in air at 450 ^{o}C for 30 mins, and the process was repeated, giving 3 layer NiO films with a thickness of 1.8 μm for

the SF5-3 cell and 1.6 μm for the P1 cell (measured with Dektak^{3}ST Surface Profile Measuring System). The resulting electrodes were submerged in 0.3 mM dye solutions in ethanol for 24 hours at room temperature.

The as-prepared counter electrode and photocathode were sandwiched together using a thermoplastic frame (Surlyn, 25 μm thickness) as a spacer. An iodine electrolyte containing LiI (1 M) and I_2 (0.1 M) in dry acetonitrile was introduced into the device via vacuum backfilling through the pre-drilled hole. Another Surlyn piece and a glass cover slip was then used to seal the cell.

Dye loading experiments were carried out by submerging NiO films in a 0.1 mM solution of the dye in DMF. Absorbance spectra were taken on the solutions over time and the difference in concentration calculated by using the dyes' molar absorption coefficient to give a mean dye loading for each film. To measure the photovoltaic parameters of devices, an Ivium CompactStat potentiostat was used in conjunction with a Xenon light source (Newport, 300 W) calibrated to 100 mW cm^{-2} with a Si diode. A black aperture was used to mask the device working area and the current was measured at applied bias between 0.1 V and -0.05 V at a rate of 5 mV s^{-1}.

Computational Methods

Optimised geometries were first calculated in vacuum, then in solvent (using the IEF PCM). Frequency calculations were used to check that the optimised geometries were true energy minima. Time-dependent DFT (TDDFT) calculations were then performed to calculate the electronic transitions. A cam-B3LYP 6-311G ++ (d,p), exchange functional and basis set combination was used.

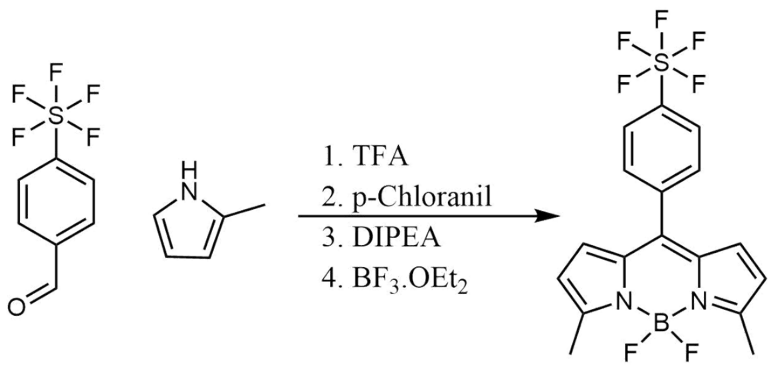

SF5-1

Dry DCM (100 mL) followed by 4-(pentafluoro--λ^6-sulfanyl)benzaldehyde (1g, 4.3 mmol), 2-methyl-1*H*-pyrrole (0.8 mL, 9.5 mmol), and three drops of trifluoroacetic acid was added to a 250 ml Schlenk tube under an inert nitrogen atmosphere. The resulting reaction mixture was stirred at room temperature for 3 hours. P-chloranil (1.27g, 5.2 mmol) was added portion wise, half in the first 5 minutes and the rest at the end of 10 minutes, following which the reaction mixture was stirred at room temperature for 3 hours under an inert nitrogen atmosphere. Diisopropyl ethyl amine (5.3 mL, 30 mmol) was added and stirred at room temperature, in the dark, for 15 minutes. Boron trifluoride diethyl etherate (5.9 mL, 47 mmol) was added and the reaction mixture stirred at room temperature overnight (~16 h). The reaction mixture washed with water (5×500ml). The organic layer was dried over Na_2SO_4 and the solvent was removed under reduced pressure. Following silica gel column chromatography (dichloromethane: Petroleum ether 40-60 °C, 2:3) the product as an orange solid (1.2 g, 2.8 mmol, 66%). mp: 242 ^oC (decompose), R_f = 0.50 (dichloromethane: Petroleum ether, 2:3), ^1H NMR (300 MHz, $CDCl_3$): δ_H 7.88 (d, *J* = 8.8 Hz, 2H,), 7.60 (d, *J* = 8.7 Hz, 2H), 6.65 (d, *J* = 4.2 Hz, 2H), 6.30 (d, *J* = 4.2 Hz, 2H), 2.66 (s, 6H). ^{13}C NMR (300 MHz, $CDCl_3$) δ 158.80, 139.34, 137.46, 134.17, 130.51, 130.08, 126.04, 125.98, 125.92, 120.07, 14.99. ^{11}B NMR (96 MHz, $CDCl_3$) δ 0.91 (t, J = 55.8 Hz). ^{19}F NMR (282 MHz, $CDCl_3$) δ 83.35 (p, *J* = 150.5 Hz), 62.82 (d, J = 150.0 Hz), -147.54 (dt, J = 65.3, 33.1 Hz). HR-MS (*m/z*) calcd for $C_{17}H_{14}{}^{10}BF_7N_2{}^{32}S$ $[M+H]^+$: 423.0859, found 423.0954.

^{1}HNMR

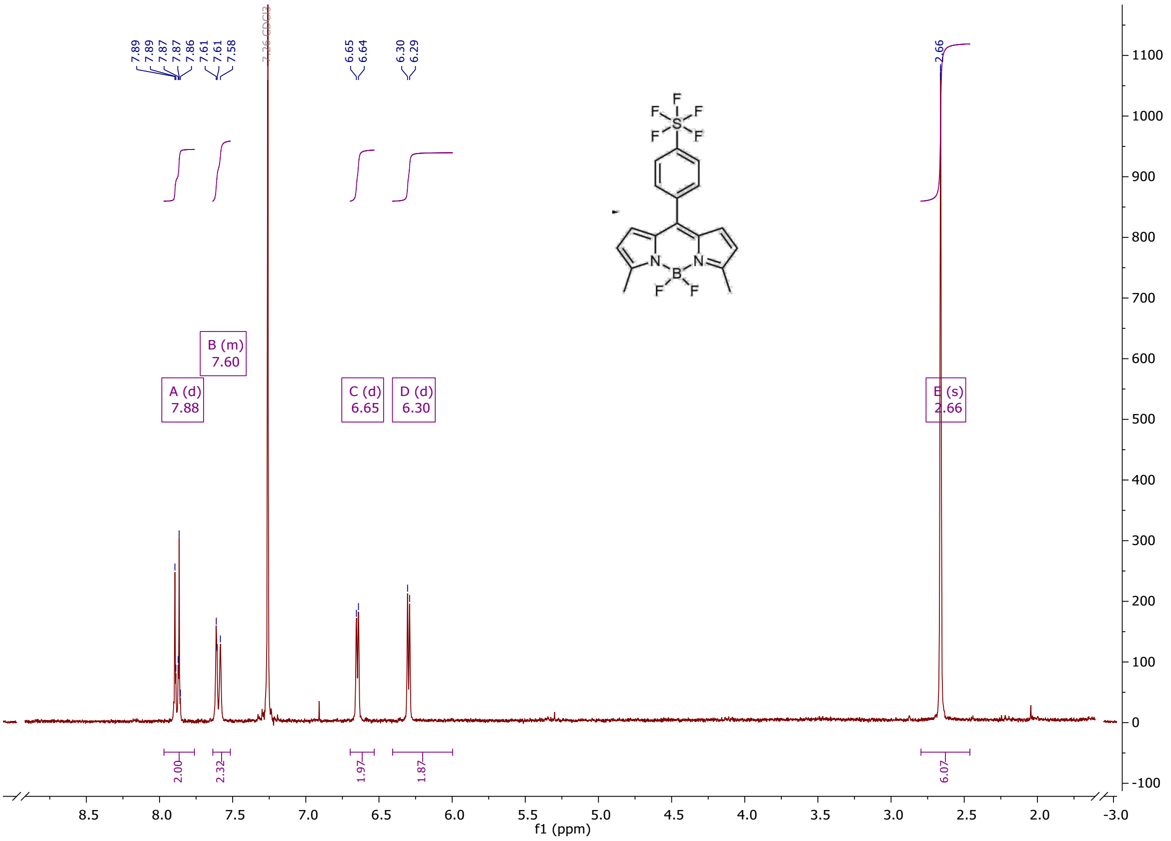

^{13}CNMR

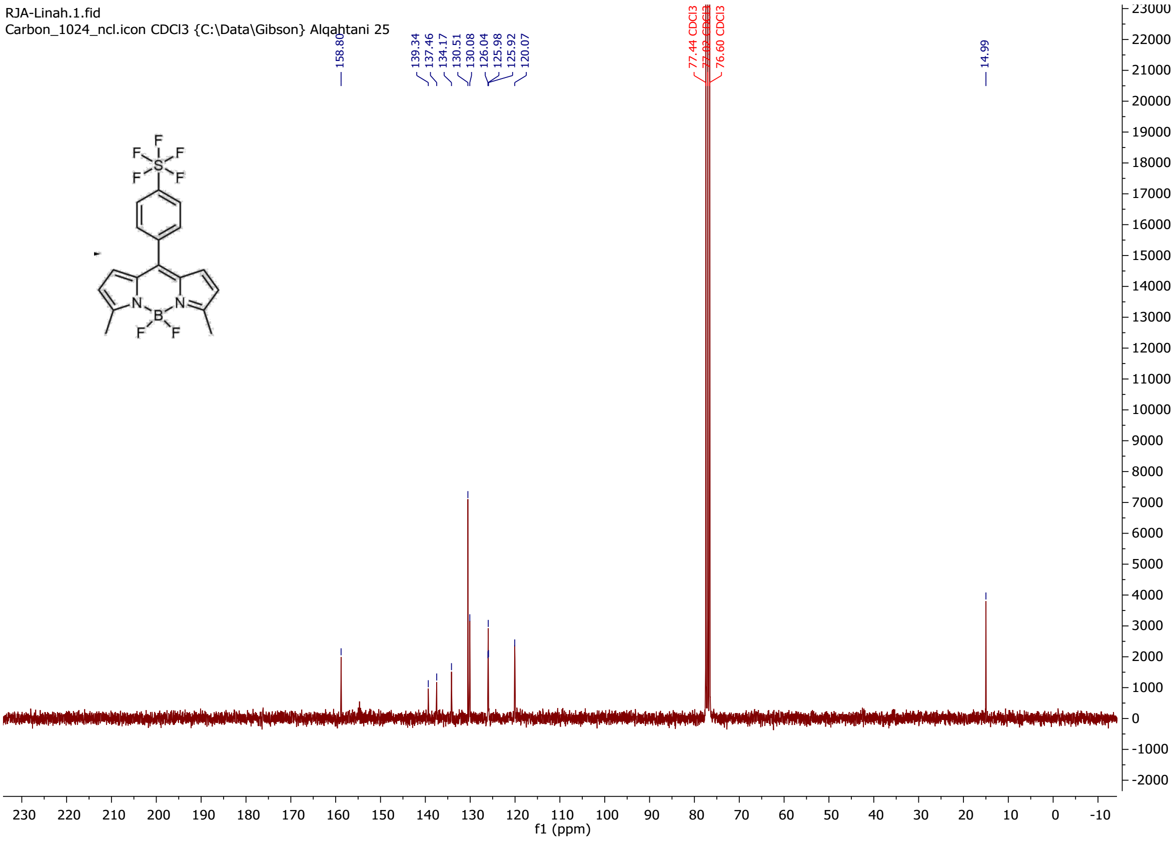

^{19}FNMR

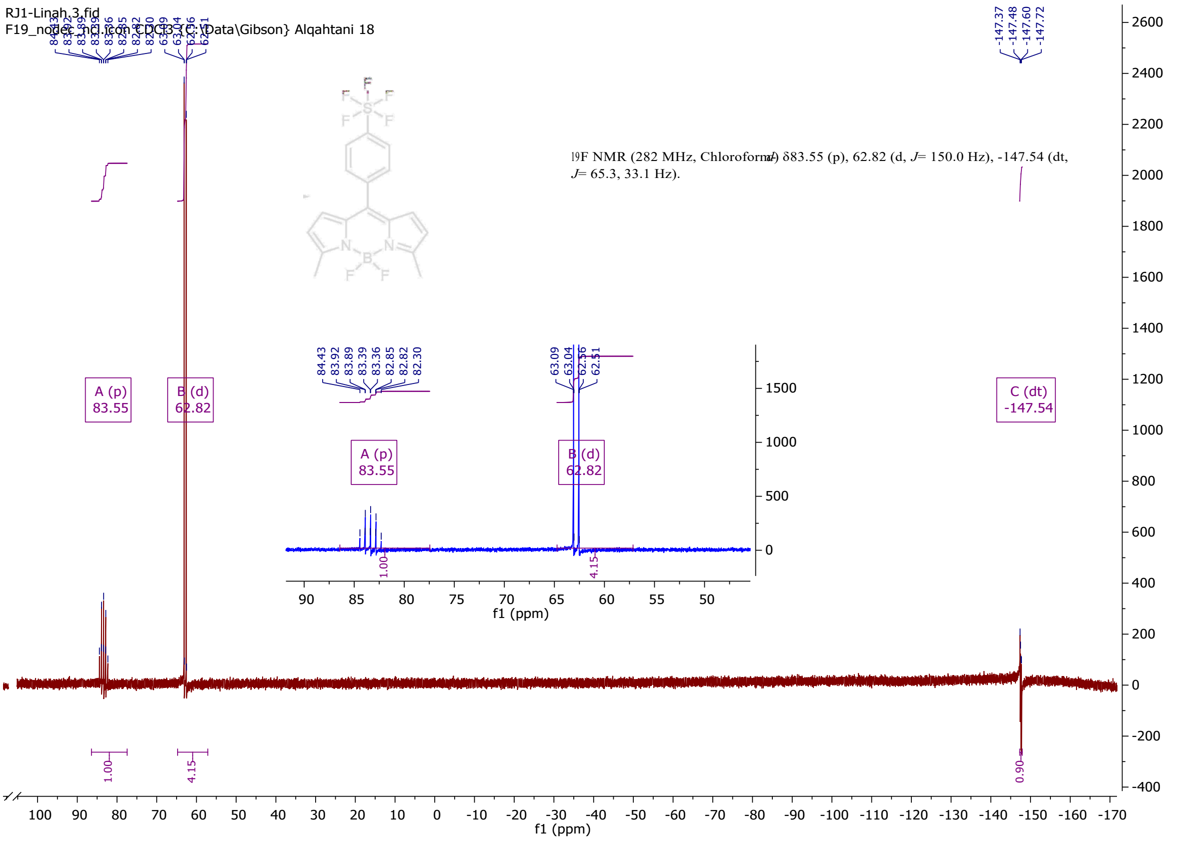

^{11}BNMR

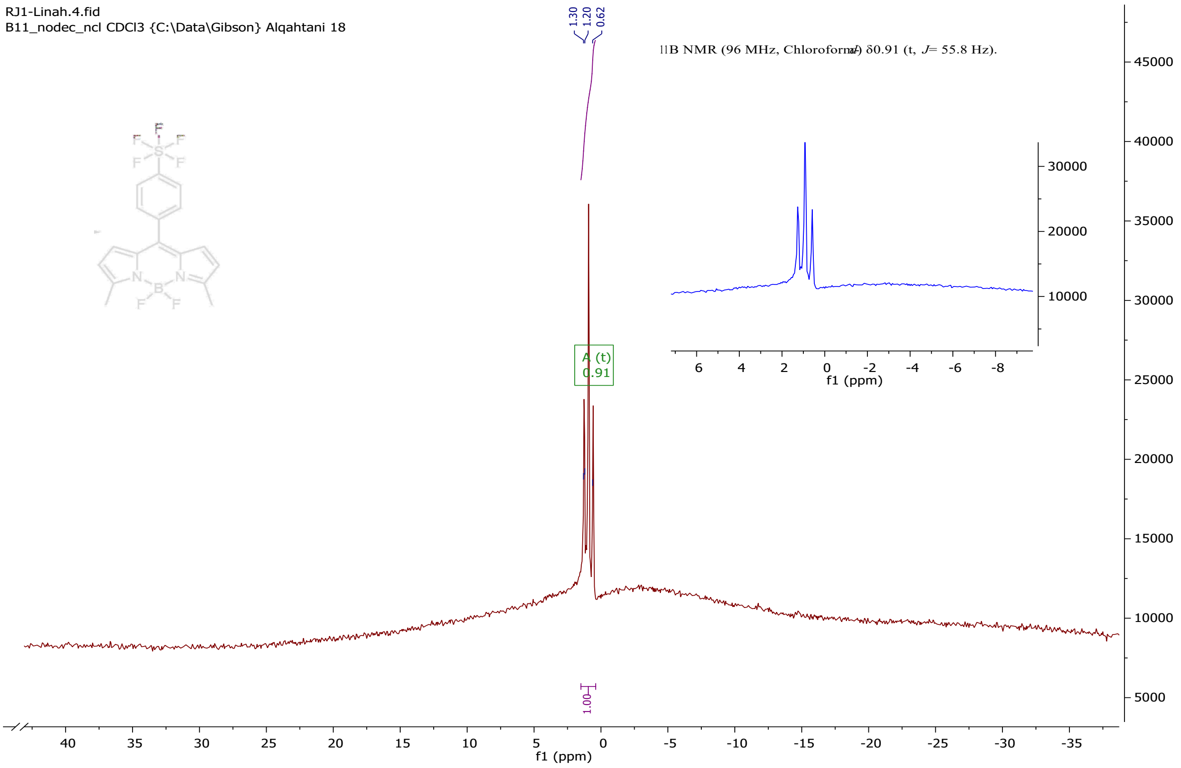

HR-MS (TOF MS)

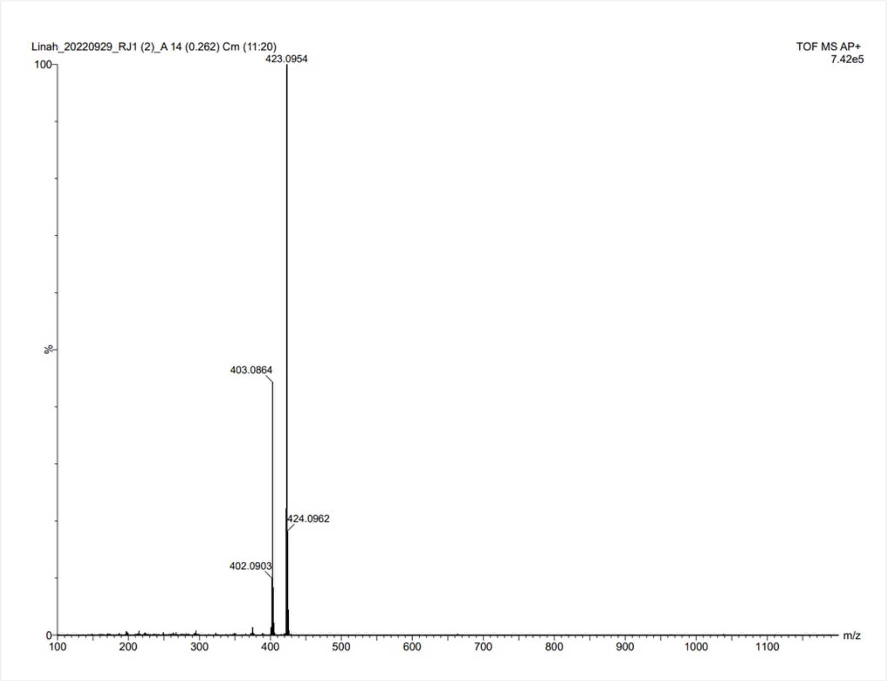

SF5-2

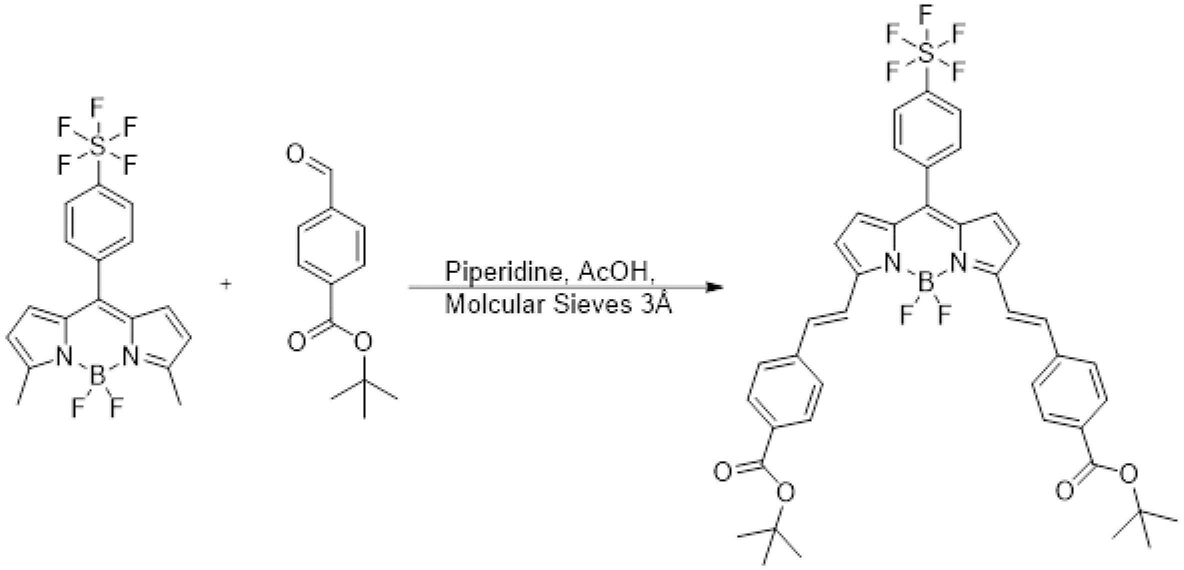

SF5-1 (500 mg, 1.18 mmol), tert-butyl 4-formylbenzoate (980 mg, 4.74 mmol), and 3Å molecular sieves (1 g) was added. to a 100 ml flame dried Schlenk under nitrogen. And degassed three times. Dry acetonitrile (20 ml) was added. Piperidine (2.34 ml, 23.69 mmol) and glacial acetic acid (1.35 ml, 23.69 mmol) were added quickly and at the same time. The

reaction was stirred at room temperature for 5 hours. The reaction was filtered, and solvent removed under reduced pressure. Following silica gel column chromatography (dichloromethane: Petroleum ether 40-60 °C, 1:1). Product was isolated as a blue solid (430 mg, 0.55 mmol, 47%). R_f= 0.37 (dichloromethane: Petroleum ether, 1:1). ^{1}H NMR (400 MHz, $CDCl_3$) δ 8.06 (d, J = 8.3 Hz, 4H), 7.95 (d, J = 8.7 Hz, 2H), 7.88 (d, J = 16.3 Hz, 2H), 7.72 (d, J = 8.3 Hz, 4H), 7.68 (d, J = 8.5 Hz, 2H), 7.42 (d, J = 16.3 Hz, 2H), 7.02 (d, J = 4.6 Hz, 2H), 6.81 (d, J = 4.5 Hz, 2H), 1.65 (s, 18H). ^{13}C NMR (75 MHz, Chloroform-d) δ 165.29, 155.26, 139.95, 136.48, 136.22, 132.36, 130.63, 129.99, 129.52, 127.37, 120.92, 117.34, 81.26, 28.23. ^{11}B NMR (160 MHz, $CDCl_3$) δ 1.47, 1.27, 1.07. ^{19}F NMR (282 MHz, $CDCl_3$) δ 83.59 (p, J = 148.1 Hz), 62.85 (d, J = 150.4 Hz), -139.19 (dd, J = 65.3, 32.0 Hz). HR-MS (*m/z*) calcd for $C_{41}H_{39}BF_7N_2O_4S$ [M- t-But]$^{+}$: 655.1461, found 655.1753.

^{1}HNMR

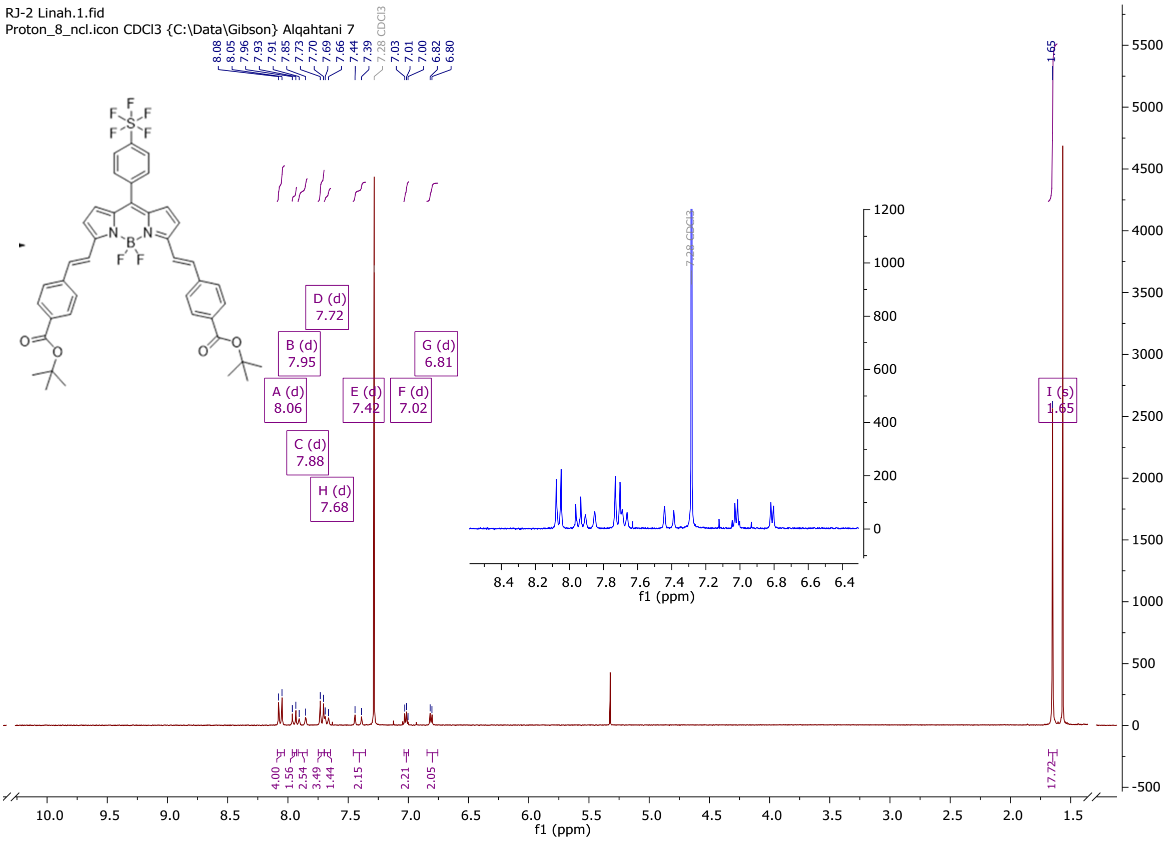

^{13}CNMR

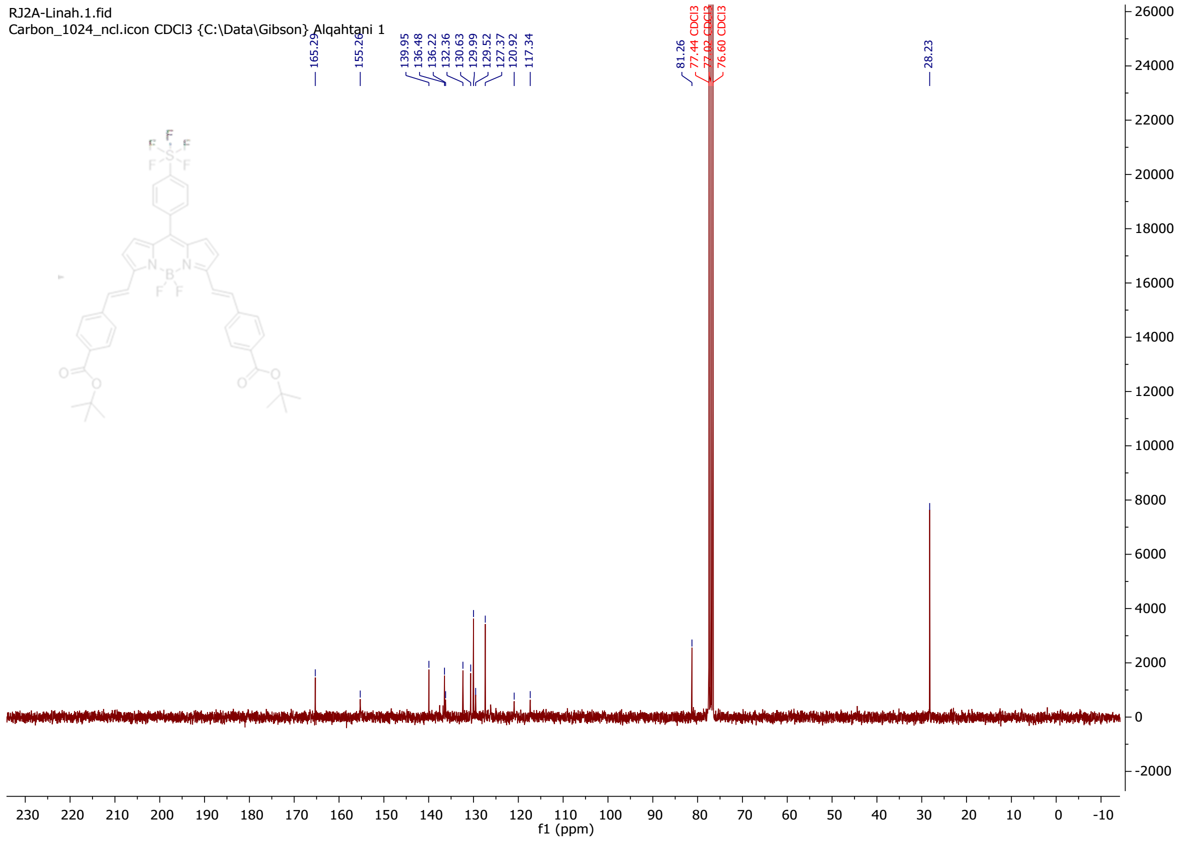

^{19}FNMR

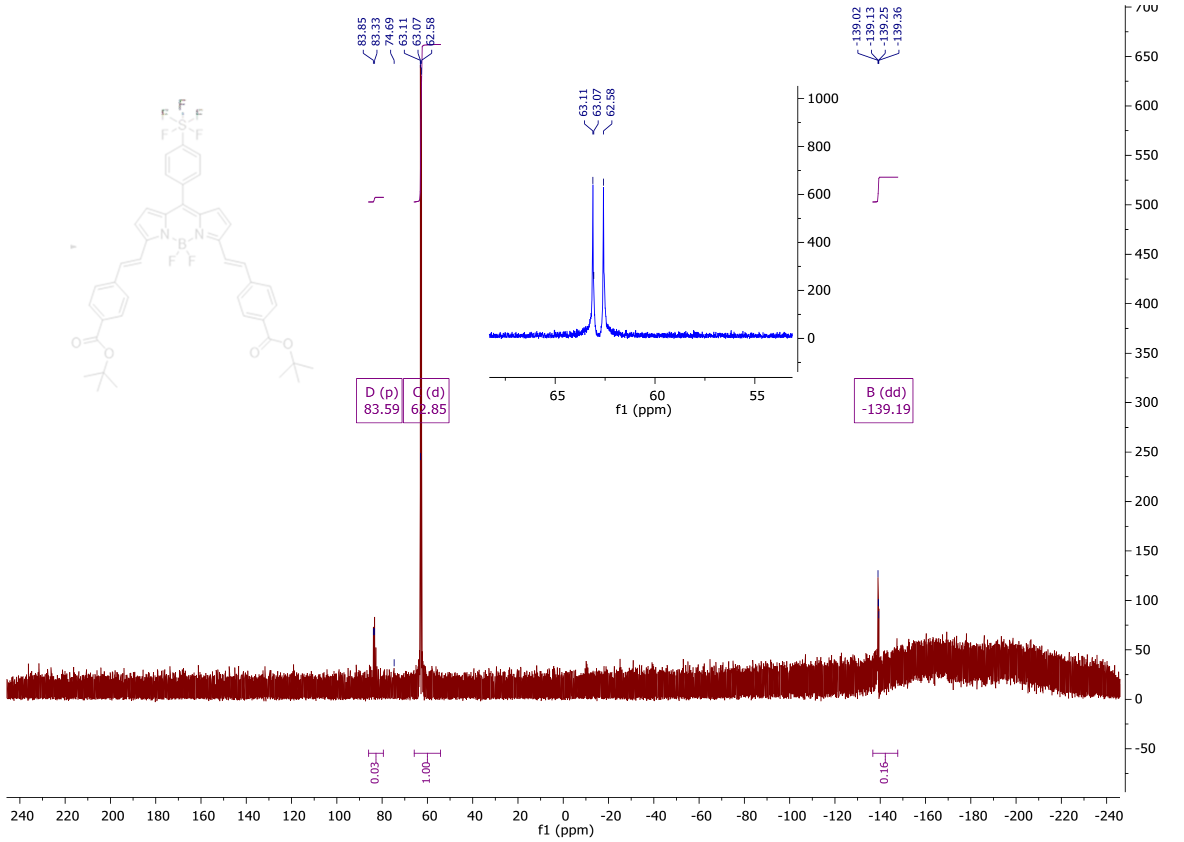

^{11}BNMR

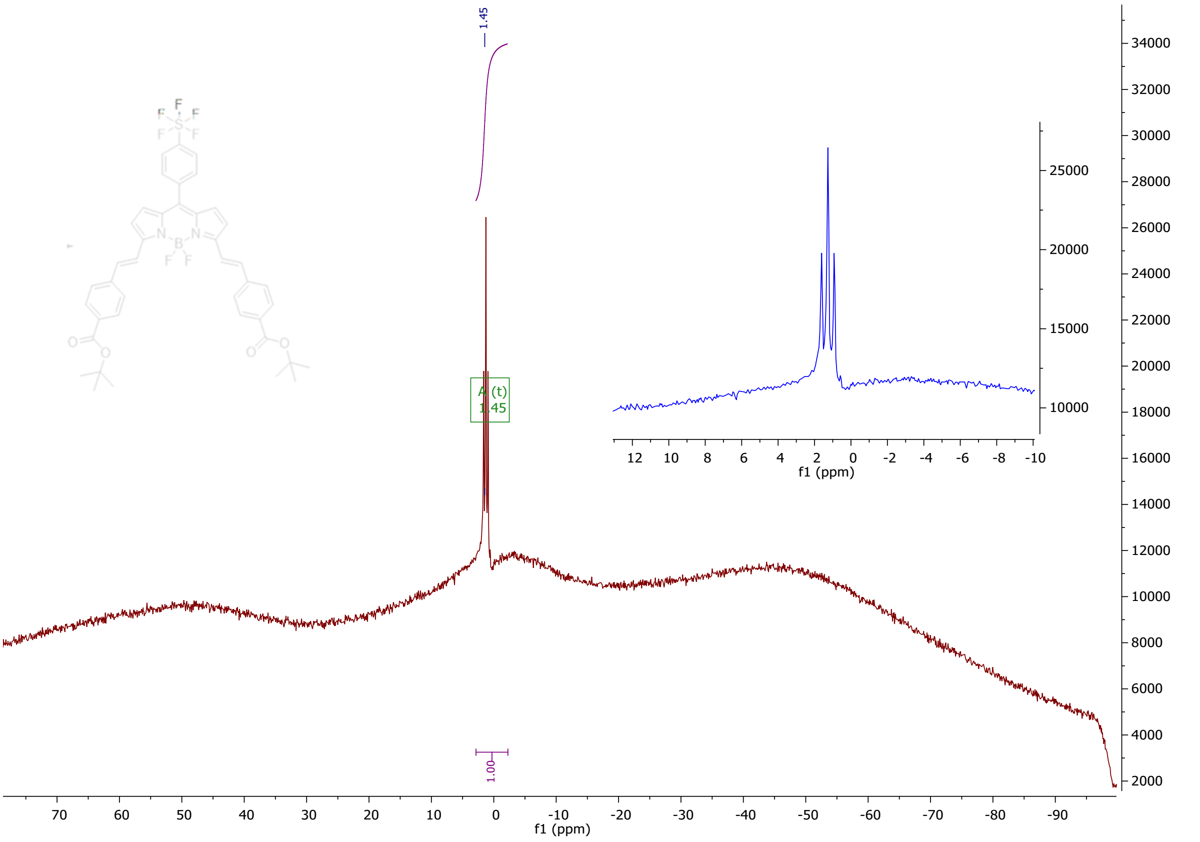

HR-MS (TOF MS)

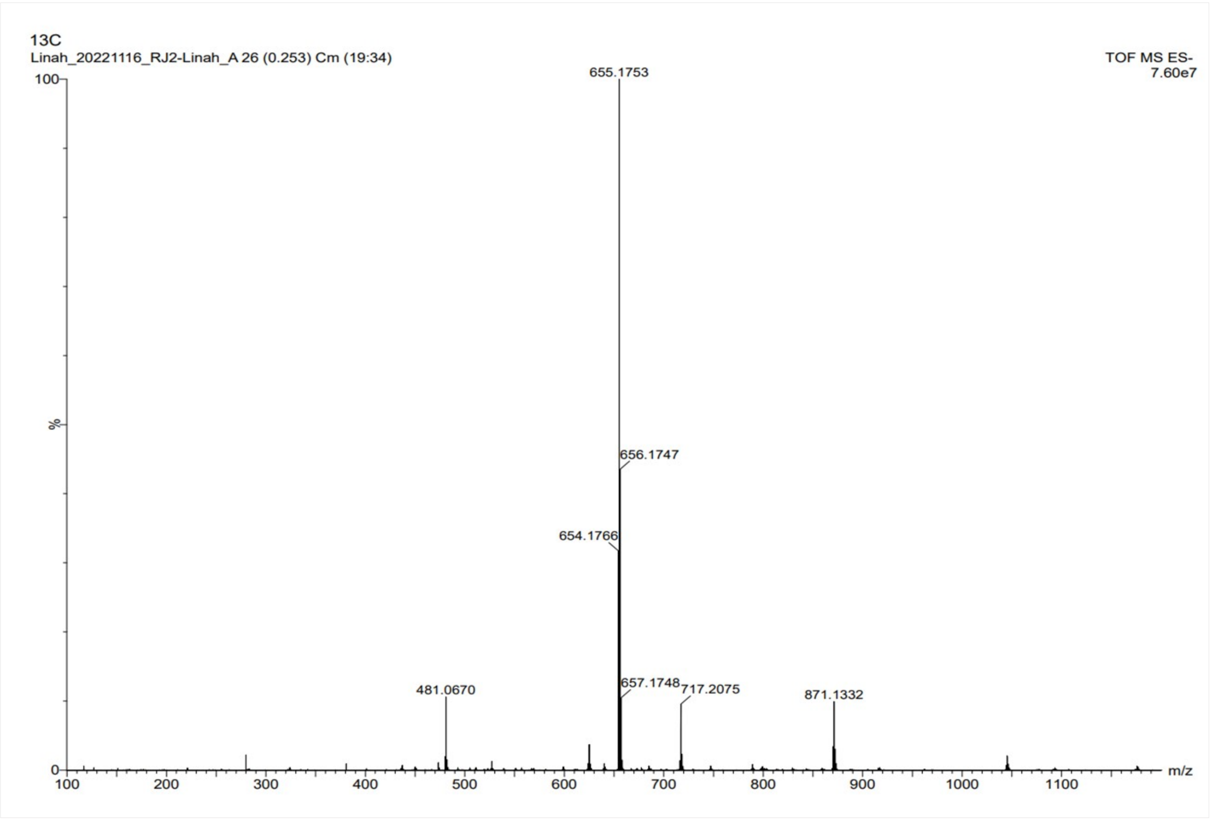

SF5-3

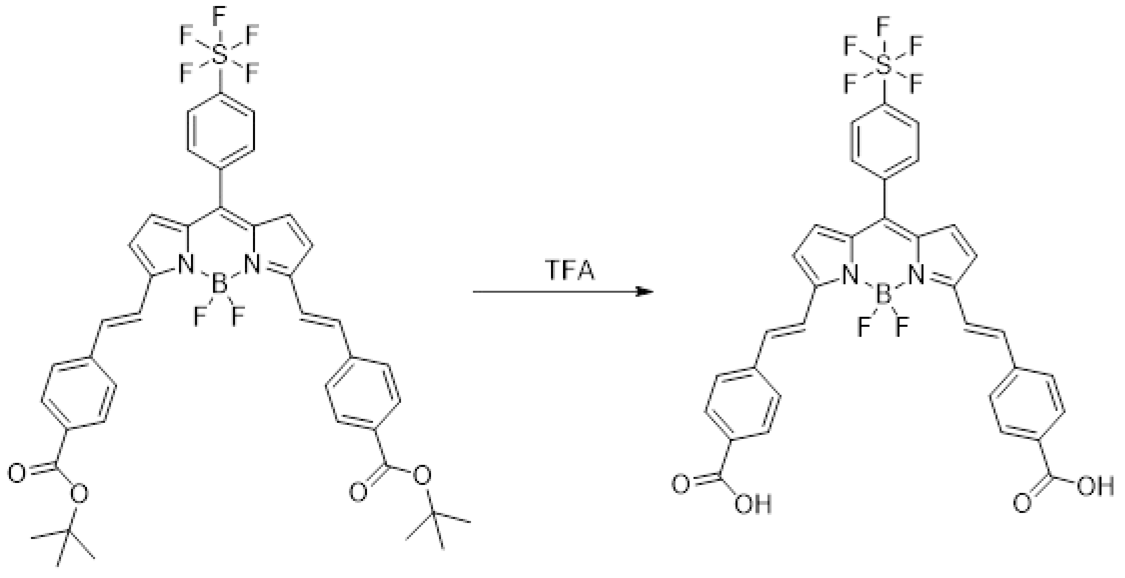

SF5-2 (50 mg, 0.070 mmol) was added to a 25 ml round bottom flask and was dissolved into 10 mL of dichloromethane. Trifluoroacetic acid (0.24 ml, 3.13 mmol) was added, and the reaction was stirred for 16 hours overnight at room temperature. TLC showed consumption of SF5-2. Solvent and TFA were removed by washing with methanol and removing the liquid by rotary evaporation followed by drying under high vacuum. The product was isolated as a blue powder (32 mg, 0.046 mmol, 73 %). $R_f = 0.82$ (THF: Petroleum ether, 1:1). ^{1}H NMR (400 MHz, THF) δ 10.86 (s, 2H), 8.08 (dd, J = 5.6, 2.8 Hz, 6H), 7.86 (d, J = 4 Hz, 2H), 7.74 (d, J = 8.0 Hz, 4H), 7.62 (d, J = 16.4 Hz, 2H), 7.21 (d, J = 4.4 Hz, 2H), 6.91 (d, J = 4.6 Hz, 2H). ^{13}C NMR (101 MHz, THF) δ 155.32, 140.42, 136.36, 131.14, 130.11, 129.47, 127.14, 126.07, 120.81, 117.35. ^{11}B NMR (128 MHz, THF) δ 1.27 (t, J = 37 Hz). ^{19}F NMR (282 MHz, THF) δ 82.40 (m), 61.11 (d, J = 149.1 Hz), -139.54 (dd, J = 65.6, 32.2 Hz). HR-MS (*m/z*) calcd for $C_{33}H_{22}BF_7N_2O_4S$ $[M-H]^+$: 685.1281, found 685.1238.

^{1}HNMR

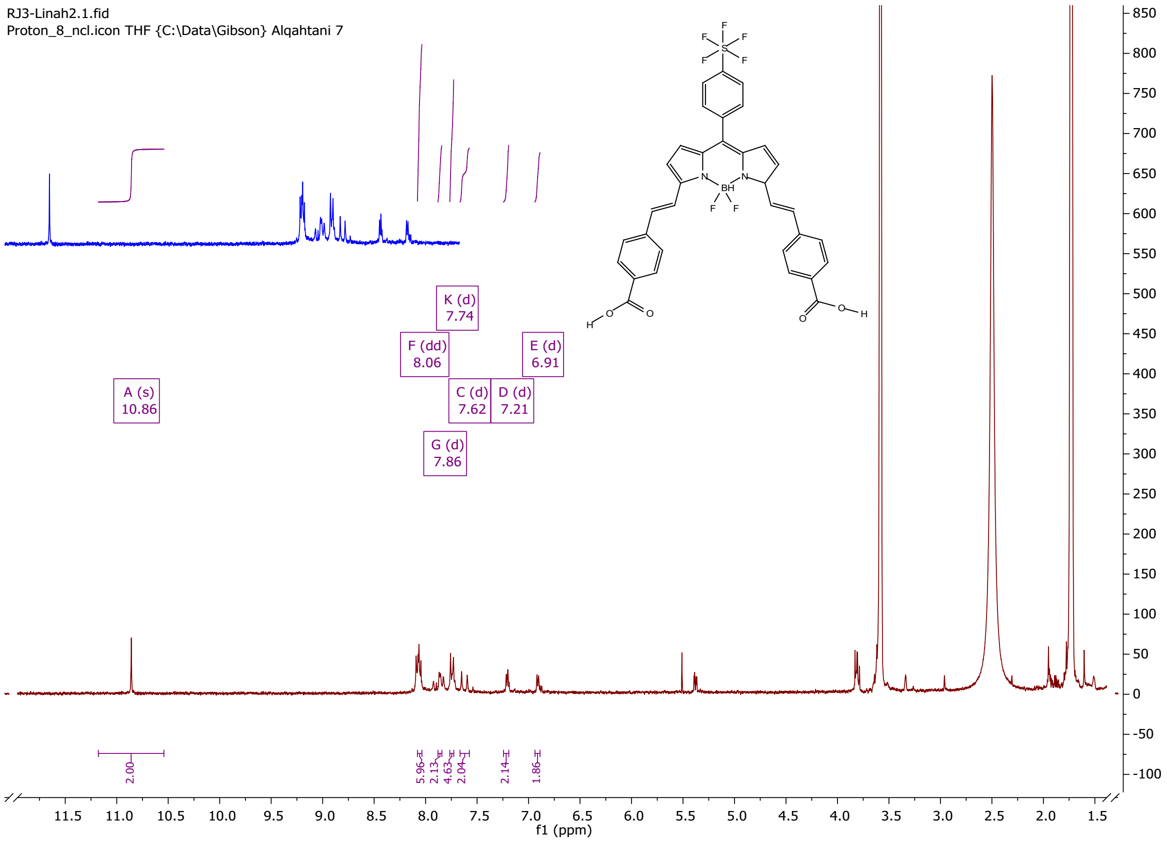

^{13}CNMR

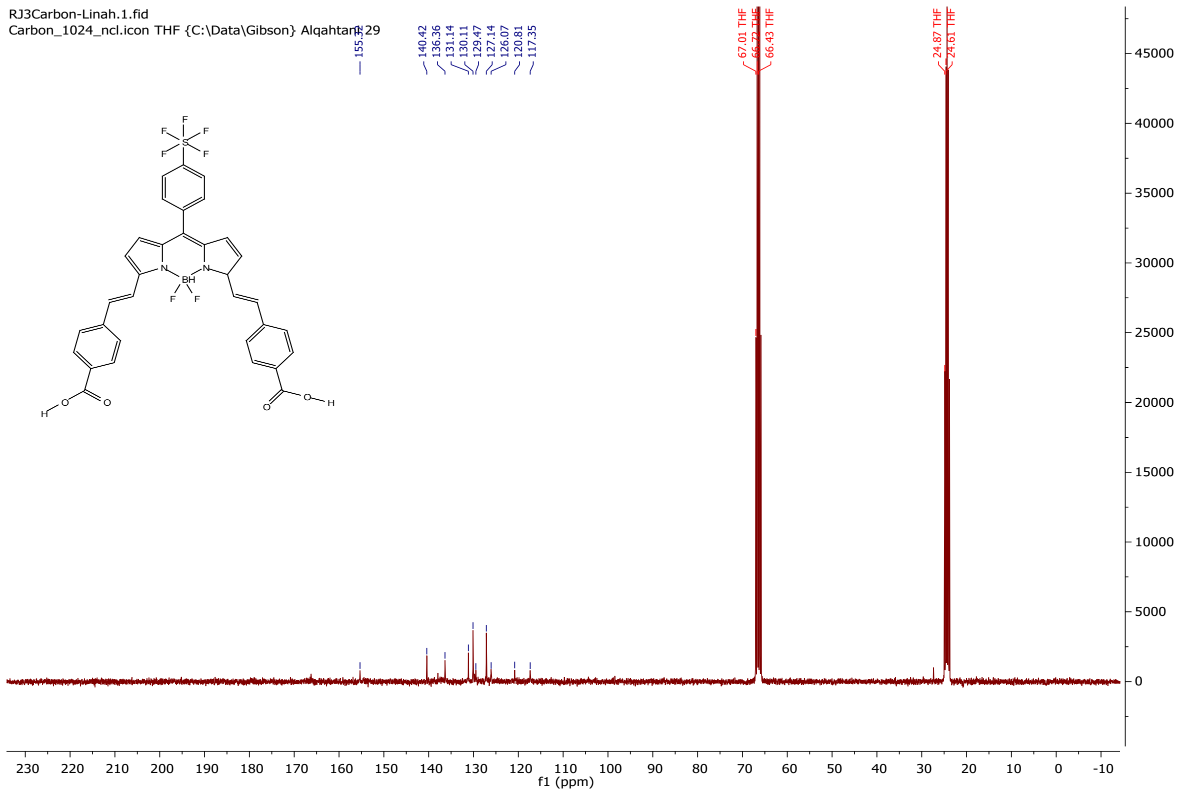

^{19}FNMR

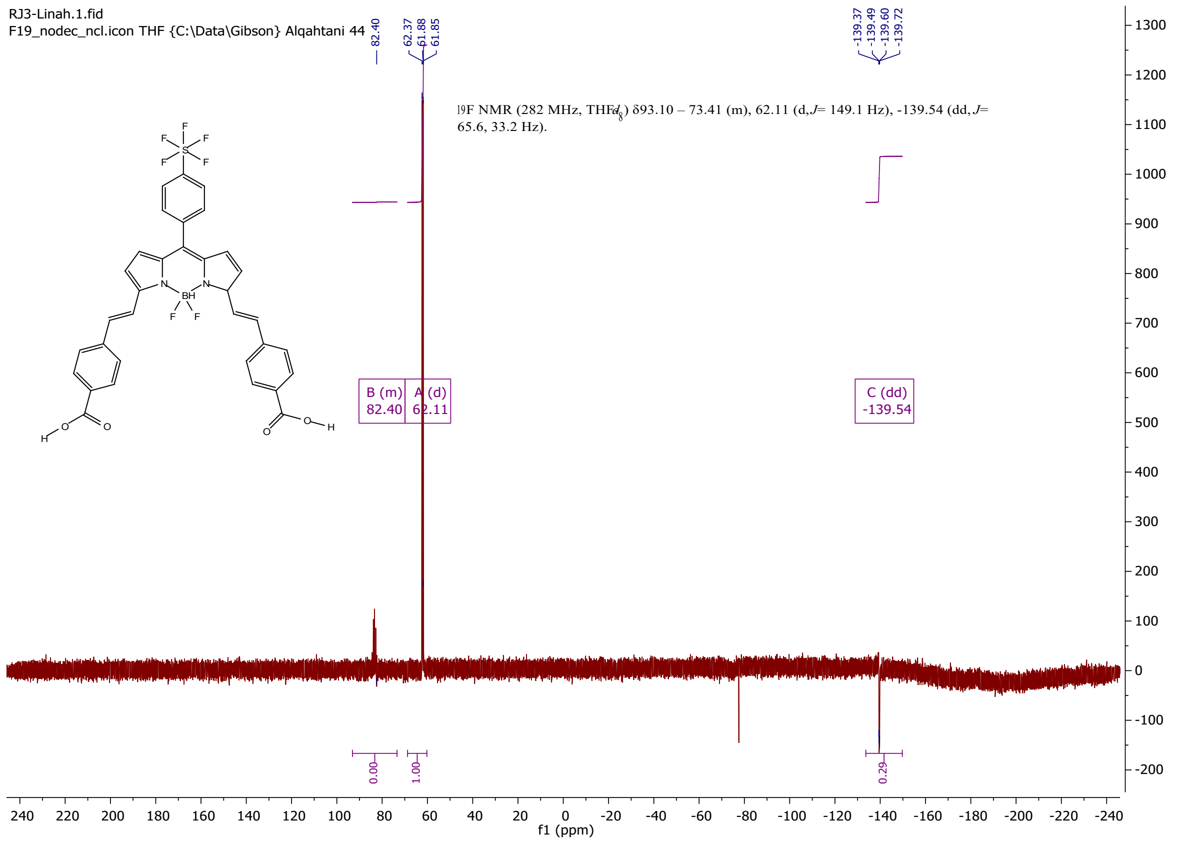

^{11}BNMR

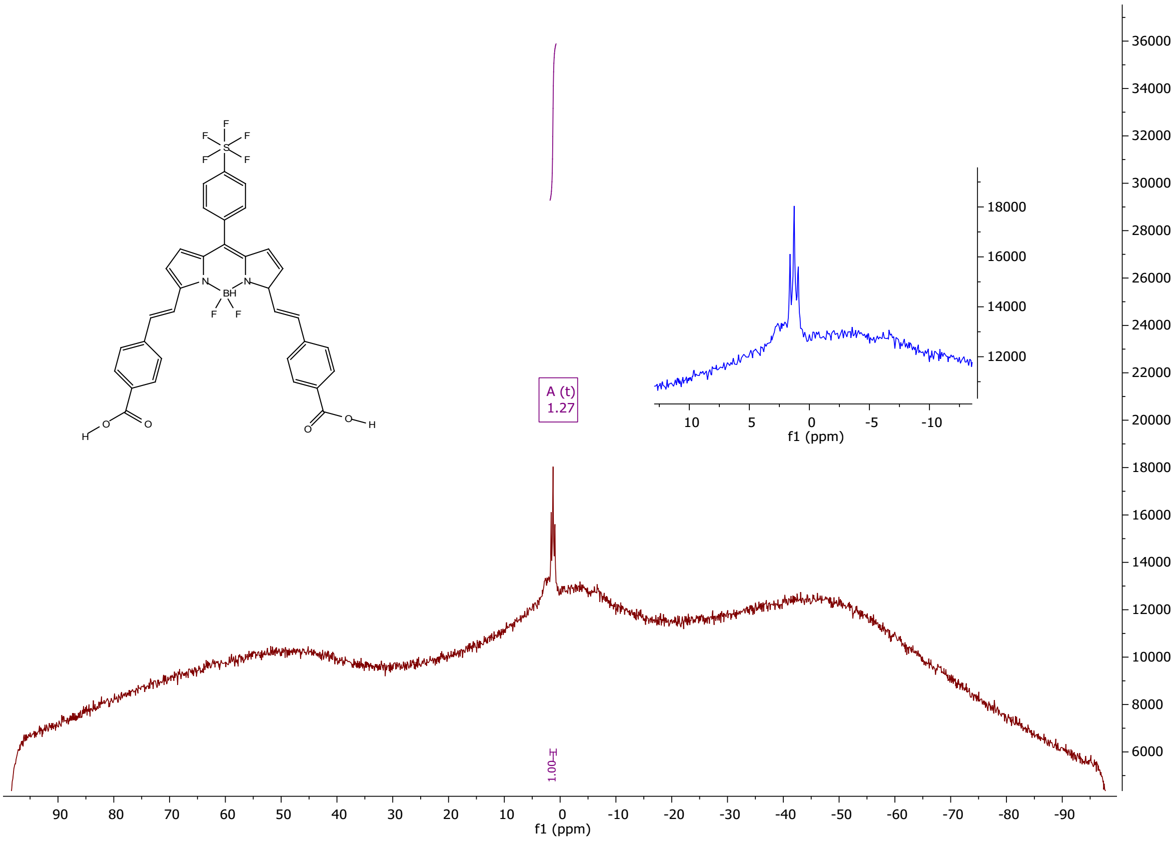
A (t)
1.27
1.00
f1 (ppm)

HR-MS (TOF MS)

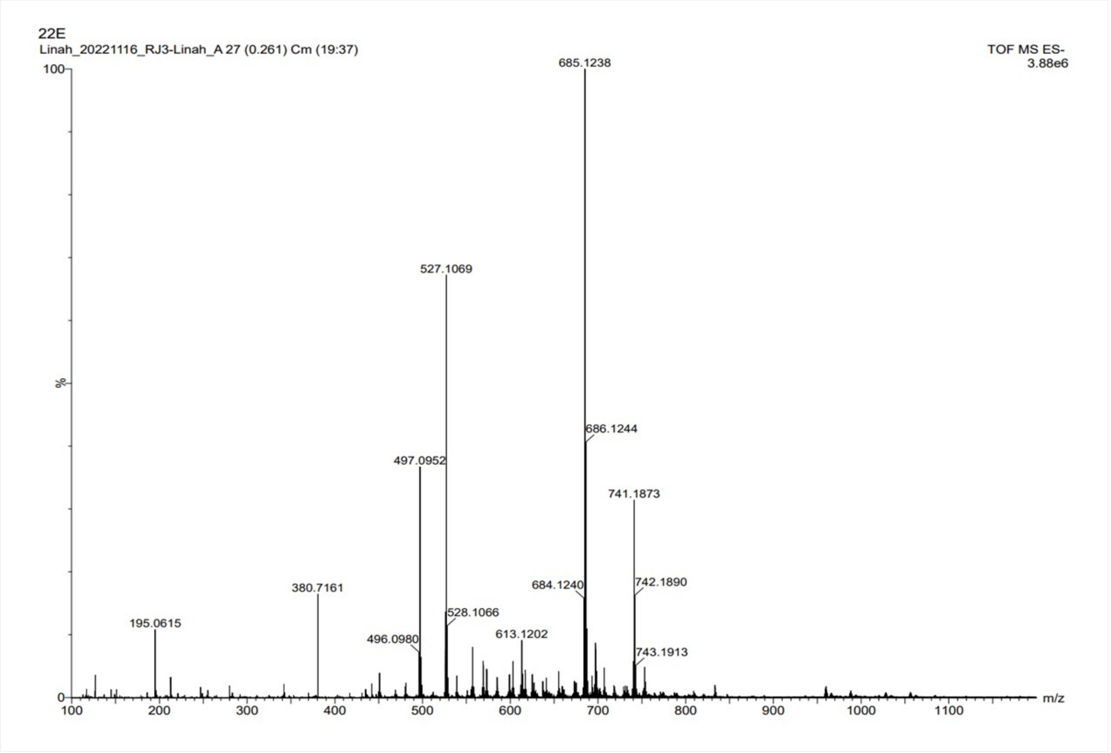
22E
Linah_20221116_RJ3-Linah_A 27 (0.261) Cm (19:37)
TOF MS ES-
3.88e6
685.1238
527.1069
686.1244
497.0952
741.1873
380.7161
684.1240
742.1890
195.0615
528.1066
496.0980
613.1202
743.1913
%
m/z

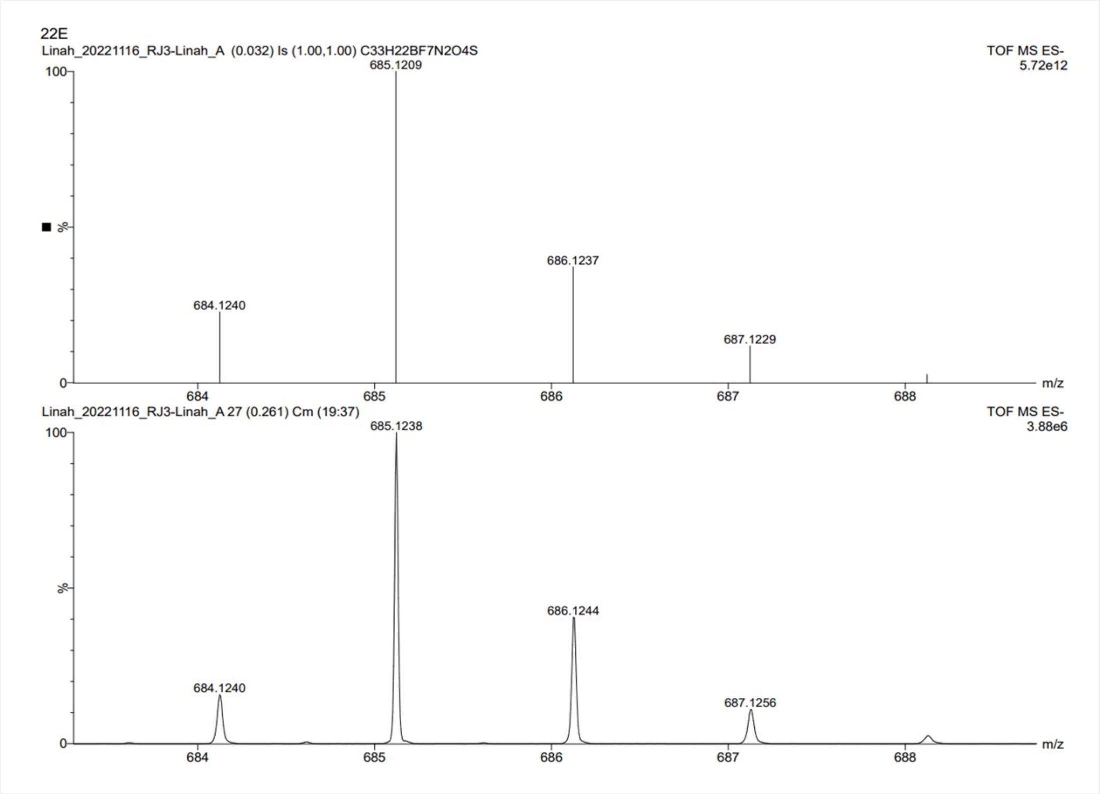

Results

Single crystal X-ray data and structure refinement

SF5-1

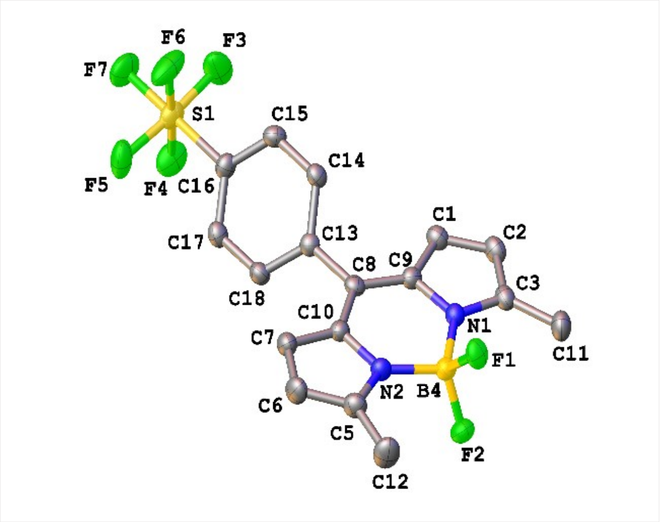

Figure S1. Crystal structure of **SF5-1**.

Table S1. Crystal data and structure refinement for SF5-1

Identification code	eag220008_fa
Empirical formula	$C_{17}H_{14}BF_7N_2S$
Formula weight	422.17
Temperature/K	150.0(2)
Crystal system	orthorhombic
Space group	Pbca
a/Å	11.6437(3)
b/Å	8.0156(2)
c/Å	36.9655(8)
α/°	90
β/°	90
γ/°	90
Volume/Å^3	3450.04(14)
Z	8
ρ_{calc}g/cm^3	1.626
μ/mm^{-1}	2.407
F(000)	1712.0
Crystal size/mm^3	0.24 × 0.06 × 0.04
Radiation	Cu Kα (λ = 1.54184)
2Θ range for data collection/°	8.976 to 154.742
Index ranges	$-14 \le h \le 13$, $-9 \le k \le 6$, $-41 \le l \le 45$
Reflections collected	12759
Independent reflections	3381 [R_{int} = 0.0302, R_{sigma} = 0.0272]
Data/restraints/parameters	3381/0/256

Goodness-of-fit on F^2	1.077
Final R indexes [I>=2σ (I)]	$R_1 = 0.0408$, $wR_2 = 0.1089$
Final R indexes [all data]	$R_1 = 0.0474$, $wR_2 = 0.1139$
Largest diff. peak/hole / e $Å^{-3}$	0.46/-0.40

Crystal structure determination of SF5-1

Crystal Data for $C_{17}H_{14}BF_7N_2S$ (M =422.17 g/mol): orthorhombic, space group Pbca (no. 61), a = 11.6437(3) Å, b = 8.0156(2) Å, c = 36.9655(8) Å, V = 3450.04(14) $Å^3$, Z = 8, T = 150.0(2) K, μ(Cu Kα) = 2.407 mm^{-1}, $Dcalc$ = 1.626 g/cm^3, 12759 reflections measured (8.976° ≤ 2Θ ≤ 154.742°), 3381 unique (R_{int} = 0.0302, R_{sigma} = 0.0272) which were used in all calculations. The final R_1 was 0.0408 (I > 2σ(I)) and wR_2 was 0.1139 (all data).

SF5-2

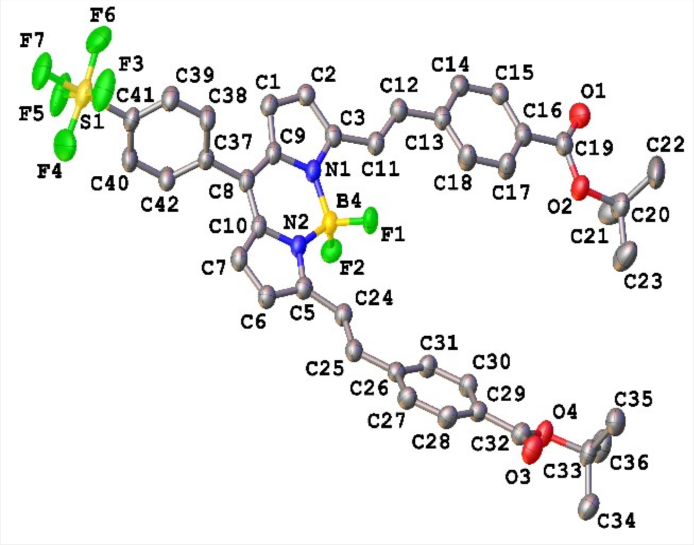

Figure S2. Crystal Structure of **SF5-2**.

Table S2. Crystal data and structure refinement for SF5-2.

Identification code	eag220009_fa
Empirical formula	$C_{41}H_{38}BF_7N_2O_4S$
Formula weight	798.60
Temperature/K	150.0(2)
Crystal system	monoclinic
Space group	$P2_1/c$
a/Å	9.4454(2)
b/Å	42.0628(8)
c/Å	11.3502(2)
α/°	90
β/°	92.954(2)
γ/°	90
Volume/Å^3	4503.44(15)
Z	4
ρ_{calc}g/cm^3	1.178
μ/mm^{-1}	1.223
F(000)	1656.0
Crystal size/mm^3	0.21 × 0.1 × 0.05
Radiation	Cu Kα (λ = 1.54184)
2Θ range for data collection/°	8.078 to 154.786
Index ranges	$-11 \leq h \leq 8$, $-52 \leq k \leq 51$, $-14 \leq l \leq 14$
Reflections collected	37821
Independent reflections	8985 [R_{int} = 0.0281, R_{sigma} = 0.0240]
Data/restraints/parameters	8985/480/511
Goodness-of-fit on F^2	1.070
Final R indexes [I>=2σ (I)]	R_1 = 0.0398, wR_2 = 0.1104
Final R indexes [all data]	R_1 = 0.0483, wR_2 = 0.1154
Largest diff. peak/hole / e Å^{-3}	0.21/-0.40

Crystal structure determination of SF5-2

Crystal Data for $C_{41}H_{38}BF_7N_2O_4S$ (M =798.60 g/mol): monoclinic, space group $P2_1/c$ (no. 14), a = 9.4454(2) Å, b = 42.0628(8) Å, c = 11.3502(2) Å, β = 92.954(2)°, V = 4503.44(15) Å^3, Z = 4, T = 150.0(2) K, μ(Cu Kα) = 1.223 mm^{-1}, $Dcalc$ = 1.178 g/cm^3, 37821 reflections measured (8.078° ≤ 2Θ ≤ 154.786°), 8985 unique (R_{int} = 0.0281, R_{sigma} = 0.0240) which were used in all calculations. The final R_1 was 0.0398 (I > 2σ(I)) and wR_2 was 0.1154 (all data).

UV-visible Absorption and Emission Spectroscopy

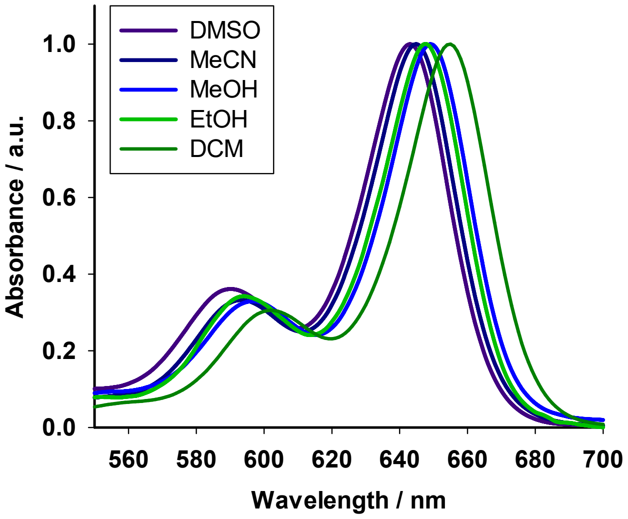

Figure S3. UV-Visible absorption spectra of **SF5-1** in different solvents.

a) *b)*

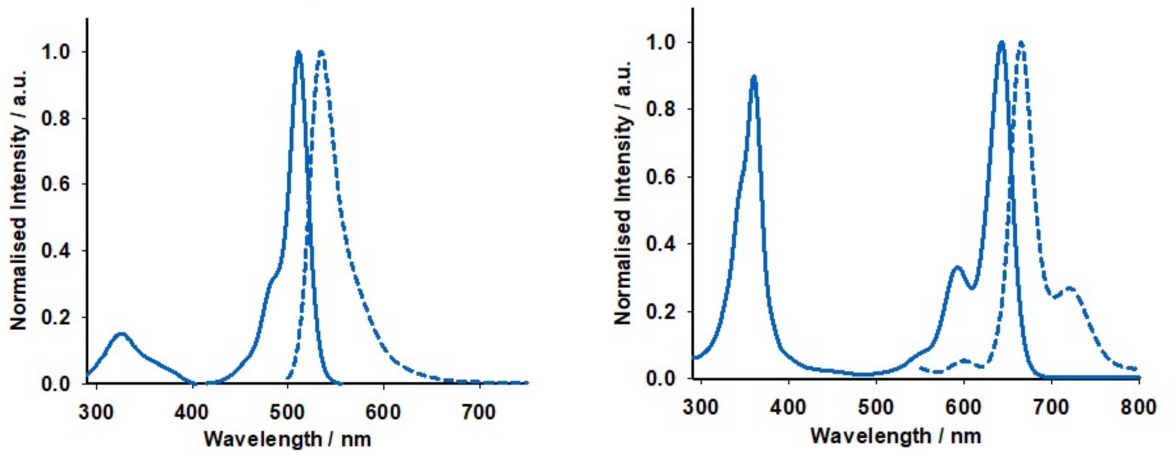

Figure S4. UV-Visible absorption and emission spectra of (λ_{ex} = 511 nm) of (a) **SF5-1** and (b) **SF5-2** in MeCN.

Photophysics

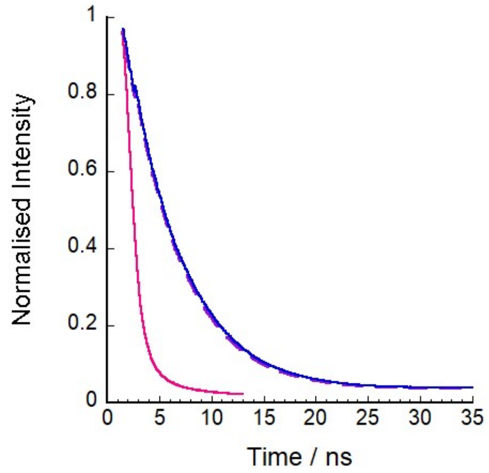

Figure S5. Emission decay of **SF5-1** (MeCN, λ_{ex} = 505 nm, magenta) **SF5-2** (purple dashed line) and **SF5-3** (THF λ_{ex} = 635 nm, blue solid line).

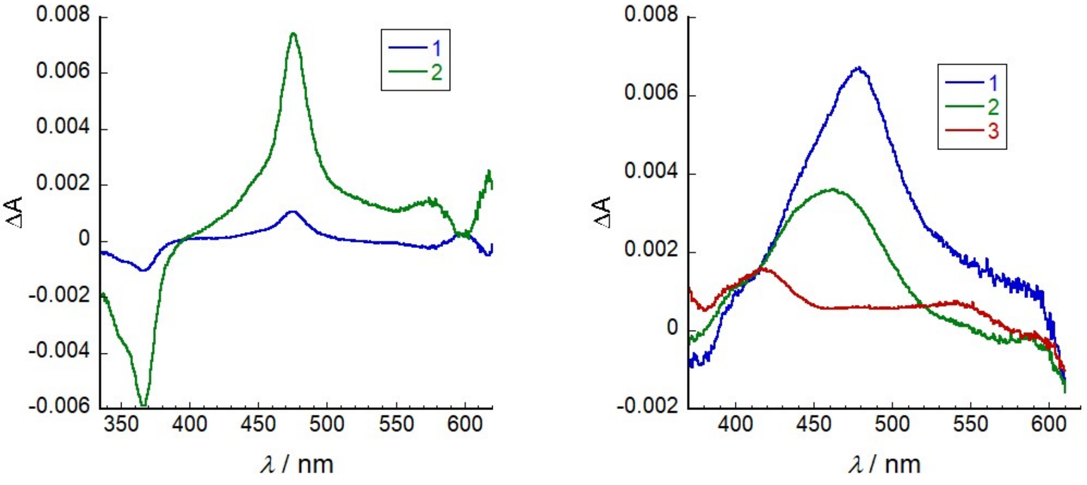

Figure S6. Decay associated spectra (λ_{ex} = 640 nm) of **SF5-3** in THF where 1 = 264 ps and 2 = 4.04 ns (left) and **SF5-3**|NiO, where 1 = 0.87 ps, 2 = 8.31 ps, 3 = 225 ns (right).

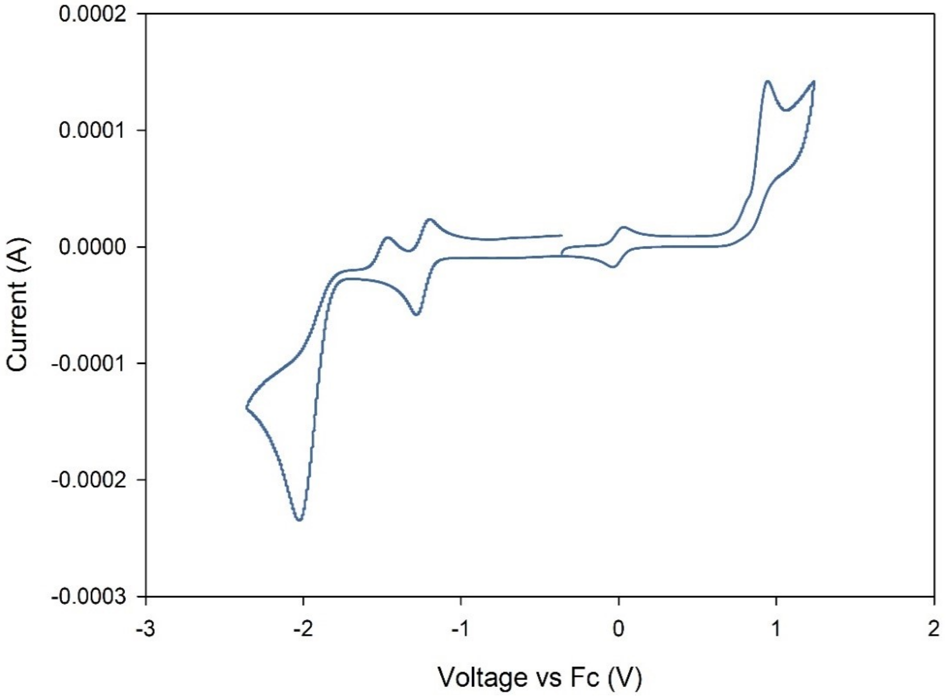

Electrochemistry

Figure S7. Cyclic voltammogram of **SF5-1** in acetonitrile with tetrabutylammonium hexafluorophosphate supporting electrolyte, using a glassy carbon working electrode and a calomel reference electrode, with scan rate of 100 mV s^{-1}.

Figure S8. Cyclic voltammogram of the first reduction for **SF5-1** at increasing scan speeds, 20, 50, 100, 200 and 500 mV s^{-1}.

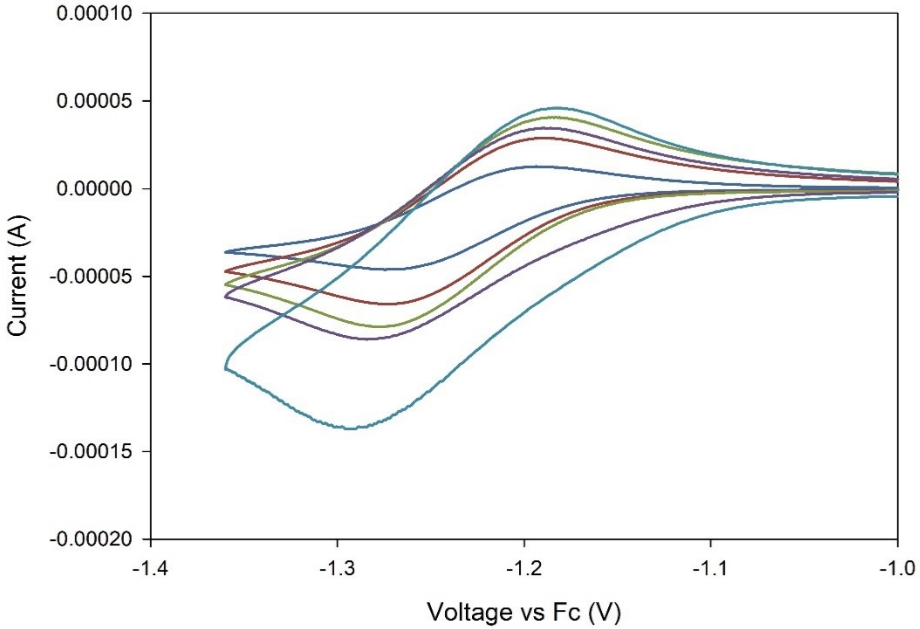

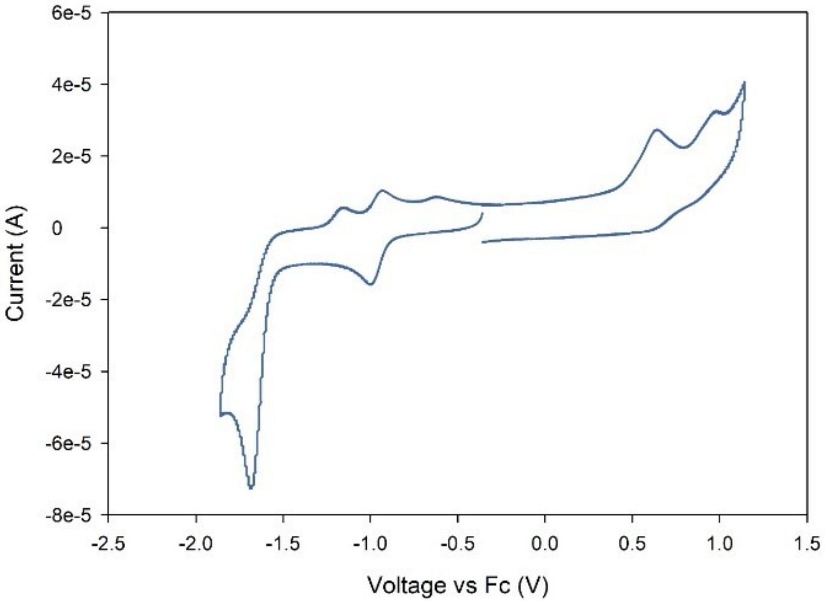

Figure S9. Cyclic voltammogram of **SF5-2** in acetonitrile with tetrabutylammonium hexafluorophosphate supporting electrolyte, using a glassy carbon working electrode and a calomel reference electrode, with scan rate of 100 mV s^{-1}.

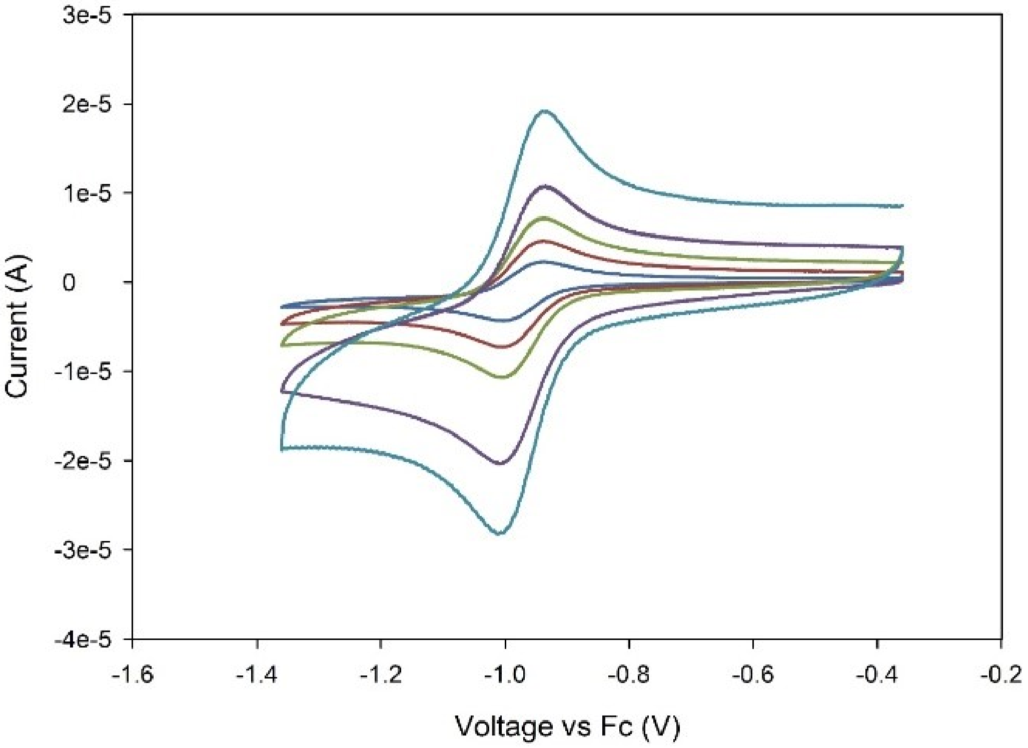

Figure S10. Cyclic voltammogram of the first reduction of **SF5-2** at increasing scan speeds, 20, 50, 100, 200 and 500 mV s^{-1}.

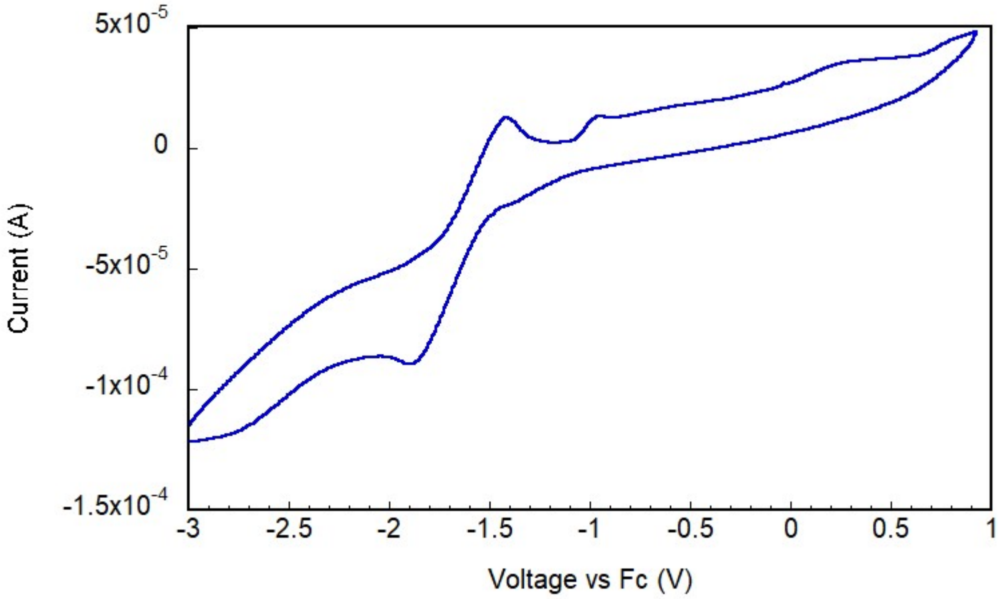

Figure S11. Cyclic voltammogram of **SF5-3** in THF with tetrabutylammonium hexafluorophosphate supporting electrolyte, using a glassy carbon working electrode and a calomel reference electrode, with scan rate of 100 mV s^{-1}.

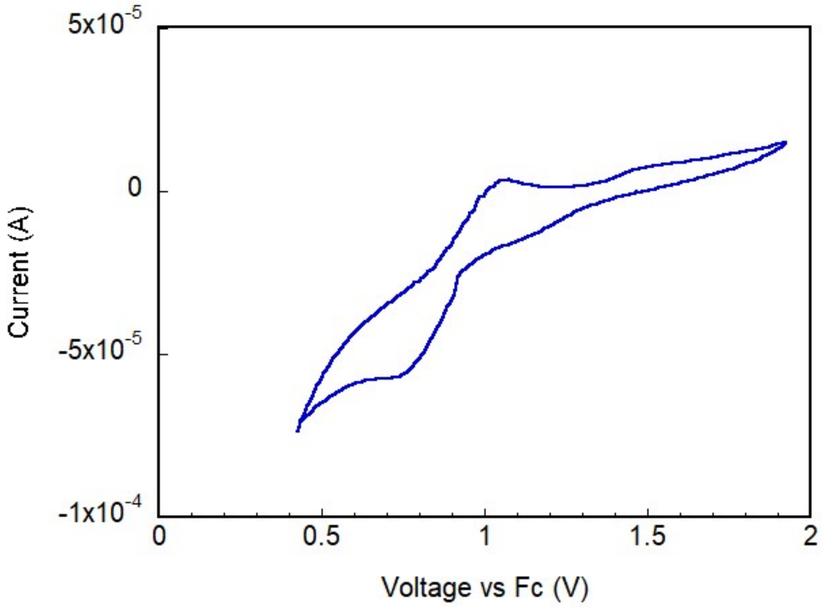

Figure S12. Cyclic voltammogram of **SF5-3** in THF with tetrabutylammonium hexafluorophosphate supporting electrolyte, using a glassy carbon working electrode and a calomel reference electrode, with scan rate of 50 mV s^{-1}.

Density Functional Theory

Table S3. Calculated singlet energy transitions for the first three excited states for **SF5-1**, and the relative contributions of each orbital transition.

Excited State	Transition	Contribution	Absorbance /nm	Energy / eV	Oscillator strength
1	HOMO to LUMO	100%	438	2.83	0.579
2	HOMO-1 to LUMO	100%	306	4.05	0.0649
3	HOMO-3 to LUMO	93%	286	4.33	0.0218
	HOMO-2 to LUMO	7%			

Table S4. Calculated singlet energy transitions for the first three excited states for **SF5-3**, and the relative contributions of each orbital transition.

Excited State	Transition	Contribution	Absorbance /nm	Energy / eV	Oscillator strength
1	HOMO to LUMO	100%	586	2.12	1.11
2	HOMO-1 to LUMO	79%	351	3.54	2.17
	HOMO to LUMO+2	21%			
3	HOMO-1 to LUMO	22%	338	3.6712	0.1365
	HOMO-1 to LUMO+3	4%			
	HOMO to LUMO+2	74%			

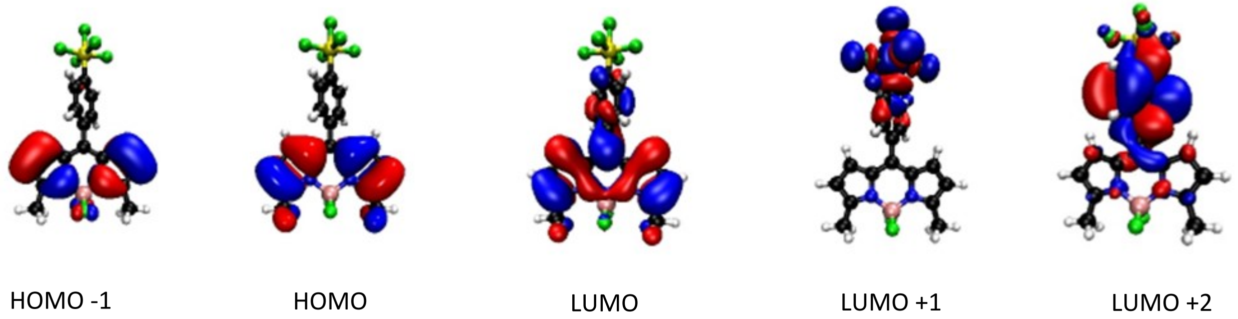

Figure S13. Frontier molecular orbitals of **SF5-1**, from left to right, HOMO -1, HOMO, LUMO, LUMO +1, LUMO +2. Orbitals rendered with Visual Molecular Dynamics software.

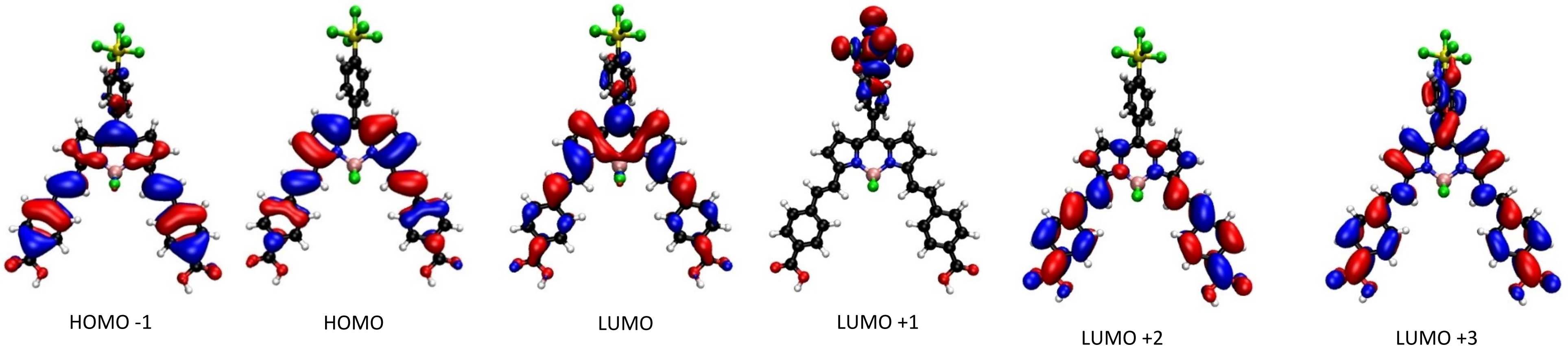

Figure 4. Frontier molecular orbitals calculated for **SF5-3**. Calculations used TD-DFT with the cam-B3LYP functional, 6-311(G) ++ (d, p) basis set. The solvent was acetonitrile using IEFPCM model

References:

1. Qin, P.; Zhu, H.; Edvinsson, T.; Boschloo, G.; Hagfeldt, A.; Sun, L. J. Am. Chem. Soc. 2008, 130, 8570–8571.
2. C. Slavov, H. Hartmann and J. Wachtveitl, *Anal. Chem.*, 2015, **87**, 2328-2336.
3. R. C. Clark, J.S. Reid, Acta Cryst., 1995, A51, 887
4. CrysAlisPro, Rigaku Oxford Diffraction, Tokyo, Japan.
5. Sheldrick, G.M. Acta Crystallogr., Sect. A: Found. Crystallogr. 2015, 71, 3-8.
6. Sheldrick, G.M. Acta Crystallogr., Sect. A: Found. Crystallogr. 2008, 64, 112-122.
7. Dolomanov, O.V.; Bourhis, L.J.; Gildea, R.J.; Howard, J.A.K.; Puschmann, H. J. Appl. Cryst. 2009, 42, 339-341.
